# Comparative transcriptome profiling and co-expression network analysis uncover the key genes associated withearly-stage resistance to *Aspergillus flavus* in maize

**DOI:** 10.1186/s12870-021-02983-x

**Published:** 2021-05-13

**Authors:** Huanhuan Liu, Haofeng Wu, Yan Wang, Huan Wang, Saihua Chen, Zhitong Yin

**Affiliations:** Jiangsu Key Laboratory of Crop Genetics and Physiology/Co-Innovation Center for Modern Production Technology of Grain Crops/Key Laboratory of Plant Functional Genomics of the Ministry of Education/Joint International Research Laboratory of Agriculture & Agri-Product Safety of the Ministry of Education, Yangzhou University, Yangzhou, 225009 China

**Keywords:** Maize, *Aspergillus flavus*, Transcriptome analysis, WGCNA, Early-stage resistance

## Abstract

**Background:**

The fungus *Aspergillus flavus* (*A. flavus*) is a serious threat to maize (*Zea mays*) production worldwide. It causes considerable yield and economic losses, and poses a health risk to humans and livestock due to the high toxicity of aflatoxin. However, key genes and regulatory networks conferring maize resistance to *A. flavus* are not clear, especially at the early stage of infection. Here, we performed a comprehensive transcriptome analysis of two maize inbred lines with contrasting resistance to *A. flavus* infection.

**Results:**

The pairwise comparisons between mock and infected kernels in each line during the first 6 h post inoculation (hpi) showed that maize resistance to *A. flavus* infection was specific to the genotype and infection stage, and defense pathways were strengthened in the resistant line. Further comparison of the two maize lines revealed that the infection-induced up-regulated differentially expressed genes (DEGs) in the resistant line might underlie the enhanced resistance. Gene co-expression network analysis by WGCNA (weighted gene co-expression network analysis) identified 7 modules that were significantly associated with different infection stages, and 110 hub genes of these modules. These key regulators mainly participate in the biosynthesis of fatty acid and antibiotics. In addition, 90 candidate genes for maize resistance to *A. flavus* infection and/or aflatoxin contamination obtained in previous studies were confirmed to be differentially expressed between the resistant and susceptible lines within the first 6 hpi.

**Conclusion:**

This work unveiled more *A. flavus* resistance genes and provided a detailed regulatory network of early-stage resistance to *A. flavus* in maize.

**Supplementary Information:**

The online version contains supplementary material available at 10.1186/s12870-021-02983-x.

## Background

Maize is a major staple food and feed crop in the world and is vulnerable to various phytopathogens. The fungus *A. flavus* can infect maize before and after harvest, leading to ear and kernel rot and subsequent contamination with aflatoxins [1]. Aflatoxins are highly toxic and carcinogenic, and mainly damage the liver tissues of humans and animals. It is estimated that 4.6–28.2% of liver cancers worldwide are caused by long-term excessive intake of aflatoxins [2]. Breeding resistant maize cultivars is regarded as the most cost-effective measure for controlling the damage of *A. flavus*. Since resistance to *A. flavus* is a complex quantitative trait [3, 4], molecular techniques, including marker-assisted selection (MAS), transgenic breeding, and gene editing, will facilitate the development of resistant cultivars. However, exploring resistance genes and gene regulatory networks is a prerequisite for the molecular breeding for maize resistance to *A. flavus*.

In the past two decades, quantitative trait locus (QTL) analysis has been widely used to map the genes conveying maize resistance to *A. flavus* infection and aflatoxin accumulation. Numerous QTLs that explained no more than 20% of the phenotypic variation using linkage mapping methods have been reported [5–13]. In addition, many single nucleotide polymorphisms associated with maize resistance have been identified through genome-wide association studies (GWAS) [14–16]. Up to now, no genes associated with *A. flavus* infection and aflatoxin accumulation in maize have been cloned through forward genetics approaches. Whereas, several genes or proteins related to maize defense have been identified and characterized by quantitative PCR (qPCR), RNA interference (RNAi), and other methods. For example, pathogenesis-related (PR) proteins, such as PR10 [17], chitinase [18], PRm [19], and lipoxygenase [20], were reported to be involved in host pathogen recognition and susceptibility to *A. flavus* infection and aflatoxin accumulation. Some proteins in the maize signaling pathway, such as calcium-dependent protein kinases, respiratory burst oxidases, and WRKY family transcription factors, function primarily in regulating the expression of antioxidant and *PR* gene expression [21]. Other factors, including phytohormones and polyamines, are also considered to be important factors in the regulation of maize resistance to *A. flavus*. For example, the increase of ethylene content in maize kernels contributed to the proliferation of *A. flavus* [22]. Further, polyamine metabolism facilitates aflatoxin resistance and overall stress tolerance in maize [23]. Though specific genes and pathways have been linked to maize resistance, a detailed description of the transcriptome dynamics and transcriptional networks during fungal infection deserves further investigation.

Transcriptome analyses based on microarray and RNA sequencing (RNA-seq) technologies can provide crucial systems-level insight into the transcriptional network of pathogen infection. Many differentially expressed genes (DEGs) and signaling pathways have been confirmed to be involved in the regulation of resistance to *A. flavus* by several maize transcriptome studies [24–27]. Recently, dual RNA-seq analyses have been used to generate gene co-expression networks, including both maize and *A. flavus* genes, to better understand the complex interaction between the two organisms [28, 29]. However, most of these studies focused on the dynamic changes of gene expression over several days after *A. flavus* inoculation, but not to the early stage of plant–pathogen interactions. Previous studies have demonstrated that the pathogen infection always triggers a rapid response in the initial stage. At as early as 3 h post inoculation (hpi), *ZmCCT*, the causal gene of a quantitative disease-resistance locus against stalk rot in maize, reached its highest expression level [30]. In the first 6 hpi, candidate genes underlying resistance to Fusarium ear rot have been identified to be differentially expressed between resistant and susceptible maize lines [31]. At 1 hpi with *Phytophthora infestans*, seven potato (*Solanum tuberosum*) pathogenesis/defense-related genes were more highly induced in the resistant cultivar than in the susceptible cultivar [32]. The dynamic changes in the early stage of *A. flavus* infection are still elusive. Therefore, it is necessary to analyze the transcriptome of maize kernels in the early stage of *A. flavus* infection.

In this study, we compared and analyzed the dynamic transcriptome reprogramming of two maize inbred lines with contrasting resistance to *A. flavus*. To identify early-stage response genes and gene co-expression networks associated with maize resistance to *A. flavus*, the kernels were sampled at 0, 0.5, 1.5, 3, and 6 hpi. Key regulatory mechanisms that determine maize resistance were identified using an integrated analysis of DEGs and co-expression networks. The expression patterns of previously discovered candidate genes that confer maize resistance were also investigated. The results of our study provide insights into the key factors and molecular mechanisms underlying the resistance of maize to *A. flavus* infection.

## Results

### Evaluation of maize inbred lines after inoculation with *A. flavus*

**Fig. 1:**
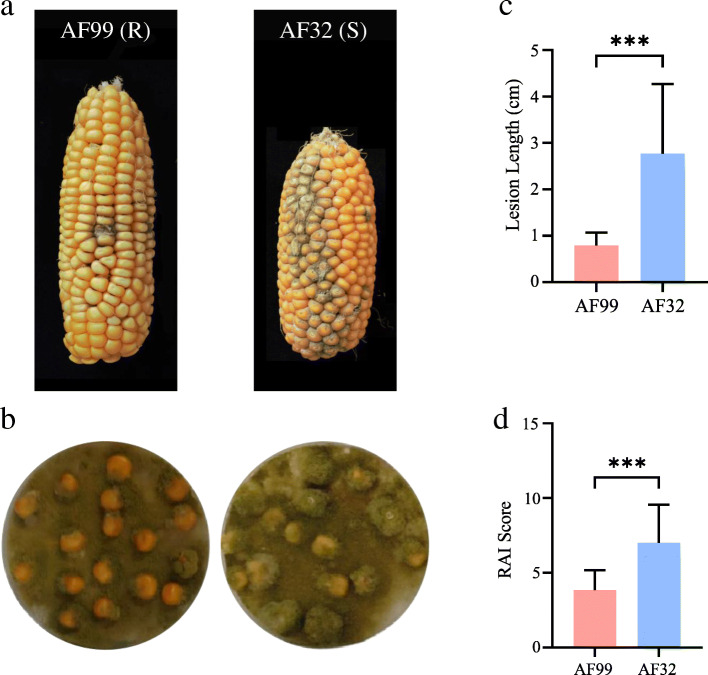
Phenotypic investigation of two maize lines at pre-harvest and post-harvest times. **a**. Harvested ears of AF99 and AF32 inoculated by *A. flavus* spore suspension at pre-harvest time (15 days after pollination). **b**. Lesion length of ears in AF99 and AF32.Values are means ± SE; *n* = 15; *** Significant difference by T-test (*P* < 0.001). **c**. Maize kernels of AF99 and AF32 co-incubated with the fungal plate at post-harvest time. **d**. RAI score of kernels in AF99 and AF32. RAI score indicates the proportion of hyphae and spores covering the kernel surface. Values are means ± SE; *n* = 60; *** Significant difference by T-test (*P* < 0.001)

Two maize inbred lines, AF99 and AF32, were selected to investigate resistance to *A. flavus* by inoculating kernels with conidia at pre- and post-harvest time points (Fig. 1). In the pre-harvest inoculation, the *A. flavus* infection was mainly restricted to the inoculated dot in AF99, while it expanded into many other kernels and resulted in a large fungal plaque in AF32 (Fig. 1a). The fungal plaque was significantly longer in AF32 than in AF99 (Fig. 1b). In the post-harvest inoculation, the fungal coverage ratio on the kernel surface (RAI score) of AF32 was significantly higher than that of AF99 (Fig. 1c, d). Hence, these two maize inbred lines vary in resistance to *A. flavus* and AF99 was more resistant than AF32 both in the pre-harvest and post-harvest inoculations.

### Global transcriptome sequencing of the two maize inbred lines

To investigate the transcriptome dynamics during the early stage of infection by *A. flavus*, we performed RNA-seq analysis on the kernels of AF99 (Resistant line) and AF32 (Susceptible line) under *A. flavus* inoculation (samples designated R and S) and ddH_2_O inoculation (samples designated RC and SC) at each time point (T0, T1, T2, T3, and T4, as described in the Methods). In total, 60 libraries were constructed and sequenced. For each library, about 6.8 Gb data were generated. After filtering low-quality reads, each library contained more than 45 million reads, 81.17% of which were mapped to the B73 reference genome (Table S1). The pairwise Pearson’s coefficients between the biological replicates were greater than 0.97, indicating a high consistency among the replicates (Figure S1). To further validate the RNA-seq data, five genes were randomly selected for real-time RT-PCR analysis (Figure S2, Table S2). The results indicated the reliability of the results of RNA-seq and the data could be used for further analysis.

### Identification of DEGs between control and infected maize kernels during infection

We conducted pairwise comparisons between mock-inoculated and fungal-inoculated kernels (R vs. RC, S vs. SC) to identify genes that respond to *A. flavus* infection at each time point in each inbred line. At the beginning stage (T0 stage), 395 (R0 vs. RC0) and 300 (S0 vs. SC0) DEGs were identified in AF99 and AF32, respectively (Table 1). These might be the initial genes that involved in the host response. Following the T0 stage, the number of DEGs was constantly changing in both lines (Table 1). These discovered DEGs were differentiated between the two lines at each time point, both in gene number and gene function (Figure S3). Only a few of these DEGs showed similar expression patterns after inoculation with *A. flavus* (5, 2, 47, 70, and 204 genes were up-regulated in both lines, and 4, 14, 7, 2, and 69 genes were down-regulated in both lines at each time point, respectively) (Figure S3), suggesting that these genes might respond to *A. flavus* infection in both lines. We also analyzed the gene expression patterns in the same genotype during infection, and found that only a few genes were differentially expressed for more than one period (Figure S4, S5). Taken together, the response of maize kernel to *A. flavus* infection was genotype-specific and infection stage-specific.

**Table 1:** The number of up‐ and down‐regulated DEGs at different time points after inoculation

Paired samples	0hpi (T0)		0.5hpi (T1)		1.5hpi (T2)		3hpi (T3)		6hpi (T4)	
	Up	Down	Up	Down	Up	Down	Up	Down	Up	Down
R vs RC	207	188	99	130	377	157	197	192	503	296
S vs SC	145	155	91	240	306	428	1243	86	838	1225
RC vs SC	3350	2341	2873	2650	4416	1963	4970	1772	3059	2615
R vs S	3206	2371	3005	2624	5075	1865	3438	2346	4001	2392

*R and RC* The resistant line AF99 was challenged with pathogen (R) or treated with water (RC), *S and SC* The susceptible line AF32 was challenged with pathogen (S) or treated with water (SC), *hpi* Hours post inoculation

**Fig. 2:**
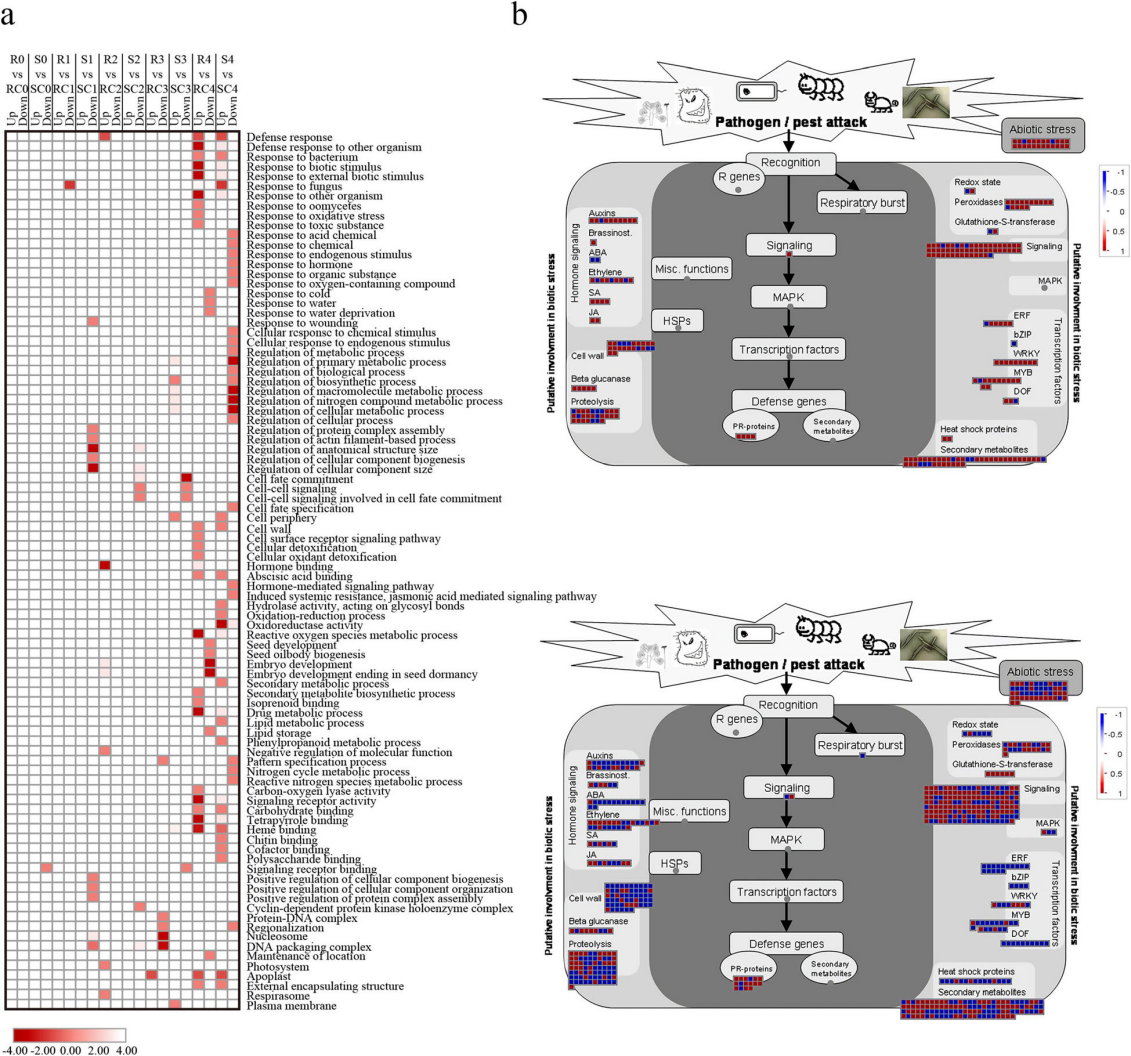
Differentially expressed genes responding to *A. flavus* inoculation in AF99 and AF32 during infection. **a**. Enriched gene ontology (GO) terms of down- and up-regulated genes responding to *A. flavus* inoculation (infection vs. mock-treatment) in AF99 and AF32 at different time points. Color depth represents the degree of significance, as shown in the scale at the bottom (corrected *p*-value was normalized). R0-R5 and S0-S5 represent samples challenged with pathogen at the 0, 0.5, 1.5, 3 and 6 hpi in AF99 and AF32, respectively; RC0-RC5 and SC0-SC5 represent samples treated with ddH_2_O at the 0, 0.5, 1.5, 3 and 6 hpi in AF99 and AF32, respectively. **b**. Biotic stress pathways visualized by searching for the known genes in response to *A. flavus* inoculation at the T4 stage in AF99 (upper panel) and AF32 (lower panel) through MapMan software. Each small square represents a gene. Blue indicates the down-regulated genes (log_2_(R4/RC4) or log_2_(SC4/S4) < − 1) and red indicates the up-regulated genes (log_2_(R4/RC4) or log_2_(SC4/S4) > 1). The scale from − 1 to + 1 represents the normalized log_2_ (fold change) in each compared group

Based on the GO analysis of the above DEGs, few terms were enriched until the T3 stage, and then numerous genes were preferentially associated with several GO terms (Fig. 2a). At the T4 stage, up-regulated genes in fungal-inoculated AF99 (R) were enriched in several different terms, which mainly related to defense responses, including “response to external biotic stimulus” (GO:0043207, corrected *p*-value = 0.0019), “response to oxidative stress” (GO:0006979, corrected *p*-value = 0.0001), “response to toxic substance” (GO:0009636, corrected *p*-value = 0.0008), “reactive oxygen species metabolic process” (GO:0072593, corrected *p*-value = 3.03E-06), “response to fungus” (GO:0009620, corrected *p*-value = 0.045), and “hormone binding” (GO:0042562, corrected *p*-value = 0.043). We also observed kernel development-related terms based on down-regulated genes, like “seed development” (GO:0048316, corrected *p*-value = 0.0001) and “embryo development” (GO:0009790, corrected *p*-value = 0.0028) (Fig. 2a). In the susceptible line AF32, up-regulated genes were enriched in relatively few pathways associated with plant defense, while down-regulated genes were also clustered into some immune-related terms, such as “oxidoreductase activity” (GO:0016491, corrected *p*-value = 0.0267), “hormone-mediated signaling pathway” (GO:0009755, corrected *p*-value = 0.0002), “response to endogenous stimulus” (GO:0009719, corrected *p*-value = 5.37E-06), and “induced systemic resistance, jasmonic acid-mediated signaling pathway” (GO:0009864, corrected *p*-value = 0.009) (Fig. 2a). KEGG analysis showed that the synthesis of some metabolites associated with plant resistance, such as benzoxazinoids, steroids, and carotenoids, was inhibited in the susceptible line after inoculation (Figure S6).

Next, we visualized the expression profiles of the DEGs in response to *A. flavus* at stage T4 via the MapMan tool. According to the general overview of the cellular response, the biotic stress response was the primary response induced (Figure S7). It was obvious that most DEGs (220/260) responding to biotic stress were up-regulated in the AF99, while only half of the genes (344/665) were up-regulated in AF32 (Fig. 2b, Table S3). Nine *WRKY* genes in AF99 (all up-regulated) and eight (half up-regulated) in AF32 were identified, and three of them (*LOC100193498*, *LOC100501702*, and *LOC103635353*) were commonly up-regulated in both lines. Most genes encoding other transcription factors, such as ERF, bZIP, MYB, and DOF, were down-regulated in the susceptible line, as well as hormone-related genes. Two-thirds of genes in the ethylene pathway were up-regulated in the resistant line AF99. Among them, *LOC103651133*, encoding ethylene response sensor 1, was up-regulated (13.9-fold). In the susceptible line AF32, half of the ethylene pathway genes were down-regulated, and the top two in the fold-change ranking were the DEAD box RNA helicase pseudogene (*LOC100272753*, 19.6-fold) and the ethylene-responsive transcription factor ERF109-like (*LOC103647485*, 16-fold). In addition, most of other genes that participate in defense pathways, like cell wall, signaling, and proteolysis, were up-regulated in the resistance line and down-regulated in the susceptible line.

### Identification of DEGs between the two maize inbred lines during infection

**Fig. 3:**
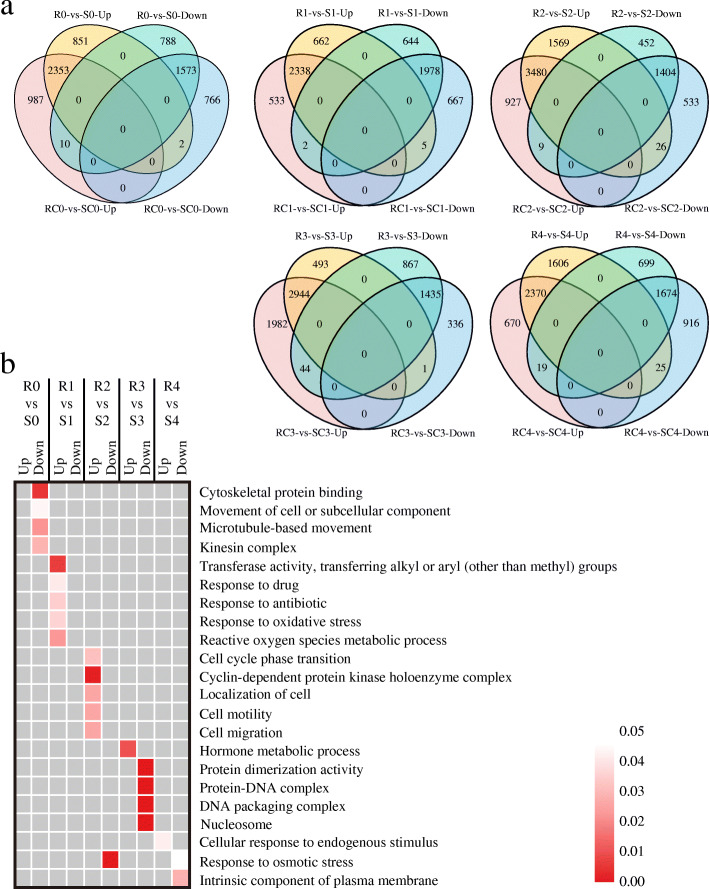
Differentially expressed genes between AF99 and AF32 with or without challenged by *A. flavus*. **a**. Venn diagrams showing the number of DEGs between AF99 and AF32 at each stage of *A. flavus* inoculation. A total number of up- and down-regulated genes (log_2_ fold change ≥ 1 or ≤ − 1 and *P* < 0.05) in AF99 as compared to AF32 in mock-inoculated (RC vs. SC) and fungal-inoculated (R vs. S) groups. **b**. Enriched gene ontology terms in the specific differentially expressed genes of fungal-inoculated kernels between AF99 and AF32. Color depth represents the degree of significance, as shown in the scale at the right (corrected *p*-value)

With or without challenge by *A. flavus*, more than 5000 genes at each stage were identified to be differentially expressed between the two lines (RC vs. SC, R vs. S), suggesting that the expression of these genes was influenced by their genetic backgrounds (Table 1). Venn diagrams revealed that a small subset of the DEGs showed differential expression only in fungal-inoculated kernels (R vs. S) (Fig. 3a), indicating that their expression difference might be induced by fungal infection. Taking the T0 stage for example, 2353 and 1573 DEGs were commonly up-regulated or down-regulated, regardless if it was inoculated with *A. flavus*. But 851 and 788 DEGs were up-regulated or down-regulated only in the fungal-inoculated kernels, respectively (Fig. 3a).

Next, we investigated the DEGs only in the fungal-inoculated kernels at the five infection stages through functional enrichment analysis. According to the GO analysis, we discovered that these up-regulated DEGs are mostly involved in plant stress responses, such as “response to drug” (GO:0042493), “response to antibiotic” (GO:0046677), “reactive oxygen species metabolic process” (GO:0072593), and “hormone metabolic process” (GO:0042445) (Fig. 3b). The KEGG analysis also suggested that these up-regulated genes were involved in different signaling pathways and biosynthesis of secondary metabolites, such as antibiotics and prodigiosin (Figure S8), whereas the down-regulated DEGs were not enriched in the defense response. These results indicate that those specific up-regulated DEGs in fungal-inoculated kernels might be involved in the immune response upon *A. flavus* challenge.

### Gene co-expression analysis during *A. flavus* infection by WGCNA

**Fig. 4:**
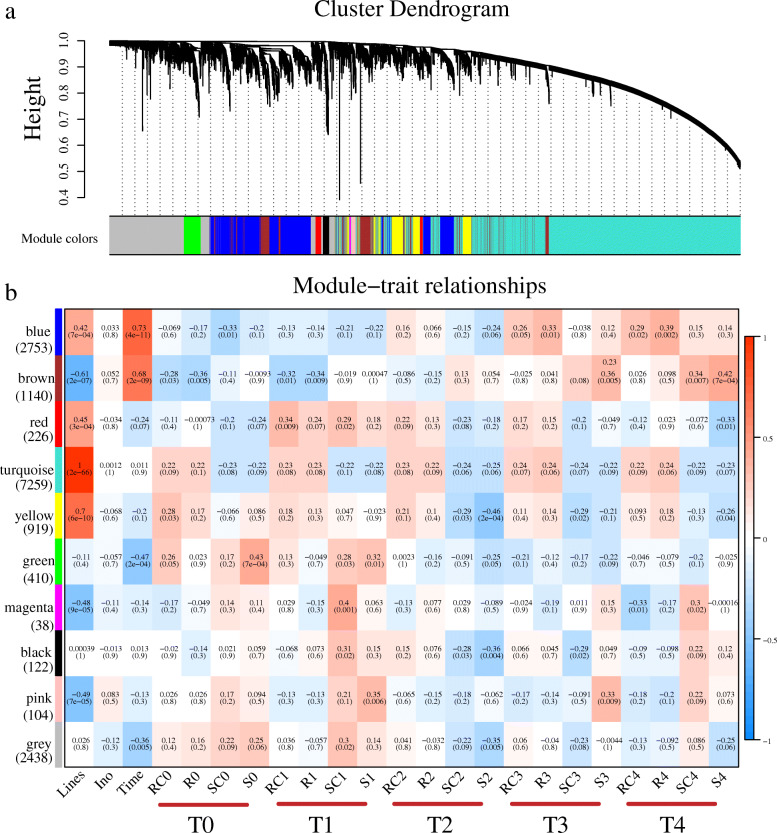
WGCNA of the transcripts in AF99 and AF32. **a**. Gene dendrograms of whole-transcriptome profiles were constructed using average linkage hierarchical clustering, each line represents one gene. The module color underneath the cluster tree shows the result of module assignment by the Dynamic Tree Cut. **b**. Correlations between modules eigengenes and lines/stages. The color of each module is the same as that in (**a**). The gene number of each module is shown in parentheses to the bottom of the module name. See the definition of phenotype in Figure S10. The correlation coefficient and *p*-value are shown in each cell

To facilitate our understanding of the regulatory network of the maize genotype-specific and infection stage-specific response to *A. flavus* infection, 60 samples and their expression data sets, including 34,315 genes, were subjected to WGCNA. Then, 15,409 genes (the first 75% of the median absolute deviation (MAD), MAD> 0.25) were selected to construct a directed network. We chose a power of β = 12 based on the scale-free topology criterion to generate a hierarchical clustering tree (Figure S9, Fig. 4a). A total of 9 co-expression modules (mergeCutHeight = 0.25) were ultimately identified, with the gene number of each module ranging from 38 to 7259. Every module was marked with different colors, and 2438 genes that did not belong to any modules were put into the grey module.

Then the correlations between the modules’ eigengenes and genotypes/stages were studied (Figure S10, Fig. 4b). Most of the modules obviously correlated with the different maize inbred lines, of which four modules (blue, red, turquoise, and yellow) showed significantly positive correlations, and three modules (brown, magenta, and pink) showed significantly negative correlation. The turquoise module, which consists of nearly half of all the genes used in the WGCNA, showed extremely high correlation to the maize inbred lines (*r* = 1, *p* = 2e-66). Notably, the expression of these genes in the turquoise module was almost impervious to fungal inoculation or infection stages (Fig. 4b), implying that they might not be involved in the resistance to *A. flavus* in the early stage.

We found that the gene expression patterns in three modules (blue, brown, and green) were significantly correlated with infection time. For instance, the expression levels of genes in the blue and brown modules were low before the T2 stage, and then gradually increased in stage T3 and T4 to various extents, while genes in the green module showed the opposite pattern. Nevertheless, no modules was identified to be associated with inoculation (mock-inoculated or fungal-inoculated, ino), which was probably due to the tremendous differences in their genetic background and rapid and dynamic changes in gene expression.

**Fig. 5:**
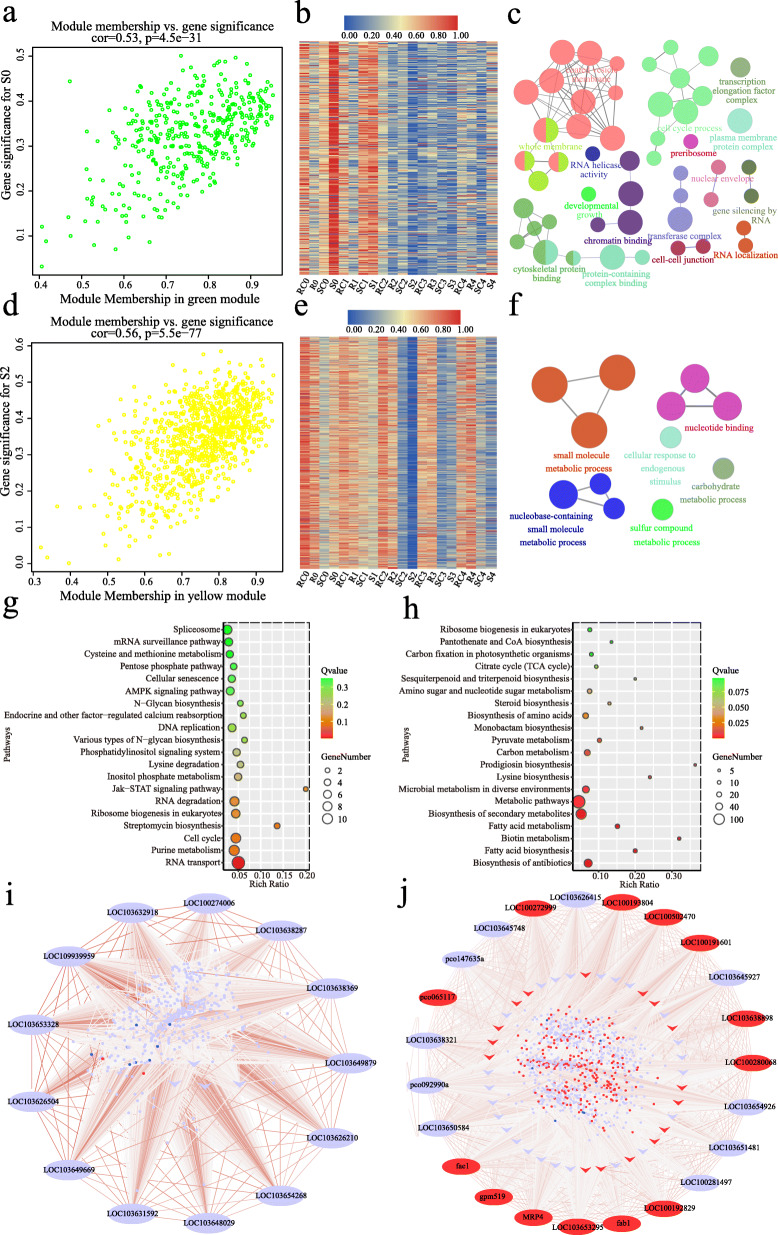
Expression profile and transcriptional regulatory network in the green and yellow modules. **a**, **d** Scatterplots of gene significance versus module membership for the green/S0 (**a**) and yellow module/S2 (**d**), with correlations and *p*-value indicated. **b**, **e** Heat map of genes in the green (**b**) and yellow module (**e**). Red indicates high expression, blue indicates low expression. The color scale represents Z-score. **c**, **f** GO analysis of genes in the green (**c**) and yellow module (**f**). Each circle represents an enrichment category, and the size of the circle indicates the number of genes. Detailed enrichment results are shown in Table S4 and S5. **g**, **h** Top 20 associated KEGG pathways for the green (**g**) and yellow module (**h**). **i**, **j** Coexpression network of the green module (**i**) and yellow module (**j**). The ellipses of the outer circle represent the hub genes, the arrows in the inner circle represent transcription factors, and the small dots in the circles represent other coexpressed genes in each module. The relationships of all the genes are connected by lines, the line color represents the weight. The red ellipse /arrow/dot indicates up-regulated (log_2_(R/S) > 1) and the blue ellipse /arrow/dot indicates down-regulated in the resistance line (log_2_(R/S) < − 1) at two spectific stages (T0 for the green module and T2 for the yellow module)

**Fig. 6:**
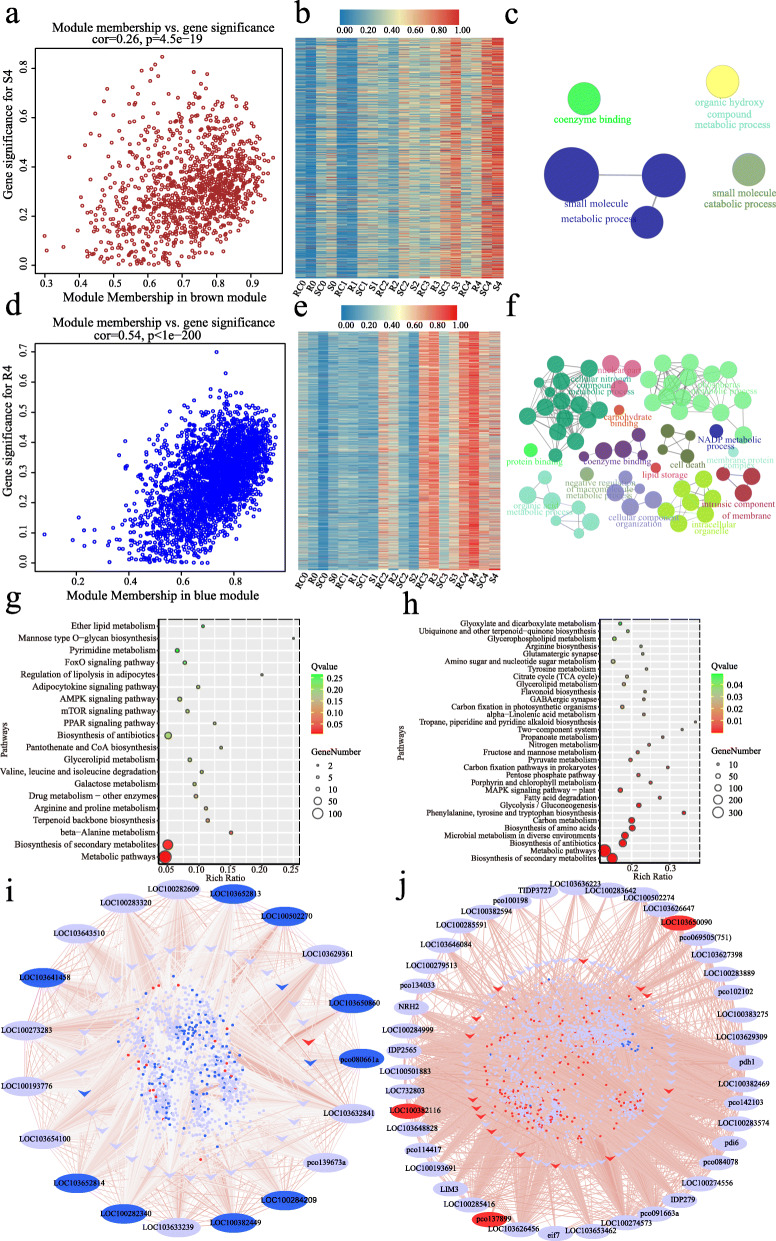
Expression profile and transcriptional regulatory network in the brown and blue modules. **a**, **d** Scatterplots of gene significance versus module membership for the brown/S4 (**a**) and blue module/R4 (**d**), with correlations and *p*-values indicated. **b**, **e** Heat map of genes in the brown (**b**) and blue module (**e**). Red indicates high expression, blue indicates low expression. The color scale represents Z-score. **c**, **f** GO analysis of genes in the brown (**c**) and blue module (**f**). Each circle represents an enrichment category, and the size of the circle indicates the number of genes. Detailed enrichment results are shown in Table S6 and S7. **g**, **h** KEGG analysis of genes in the brown (**g**, top 20) and blue module (**h**, corrected *p*-value < 0.05). **i**, **j** Coexpression network of the brown module (**i**) and blue module (**j**). The ellipses of the outer circle represent the hub genes, the arrows in the inner circle represent transcription factors, and the small dots in the circles represent other coexpressed genes in each module. The relationships of all the genes are connected by lines, the line color represents the weight. The red ellipse /arrow/dot indicates up-regulated (log2(R/S) > 1) and the blue ellipse /arrow/dot indicates down-regulated in the resistance line (log2(R/S) < − 1) at the T4 stage

To investigate the precise gene regulatory network during infection, we performed WGCNA by connecting gene co-expression modules to the four groups (R, RC, S, SC) in the successive infection stages. In general, 7 modules (blue, brown, green, yellow, black, pink, and magenta) were found to be associated with specific infection stages (Fig. 4b). At the T0 stage, the green module (410 genes) was positively correlated with S0 (*r* = 0.43, *p* = 7e-04), while the brown module (1140 genes) had a markedly negative relationship with R0 (*r* = − 0.36, *p* = 0.005). At the T1 stage, the magenta module (38 genes) and the pink module (104 genes) were positively correlated with SC1 (*r* = 0.43, *p* = 0.001) and S1 (*r* = 0.35, *p* = 0.005), respectively. At the T2 stage, both the yellow module (919 genes) and the black module (122 genes) had significant negative correlations with S2. At the T3 and T4 stages, the brown module was positively correlated with S3 (*r* = 0.36, *p* = 0.005) and S4 (*r* = 0.44, *p* = 7e-04), while the blue module (2753 genes) was positively correlated with R4 (*r* = 0.39, *p* = 0.002) (Fig. 4b). The significant correlations between stage-specific gene expression and module membership (MM) in each module were shown (Fig. 5a, d, Fig. 6a, d, and S11). Four key modules (gene number > 400) were further analyzed in the following sections.

### Characteristics and hub genes of the transcriptional regulatory modules correlated with different infection stages

In the green module, genes were up-regulated in fungal-inoculated AF32 (S) at the T0 stage compared with mock-inoculated group (Fig. 5b). According to the GO analysis, we obtained 50 significant GO terms (corrected *p* < 0.05), and the top two terms were “plasma membrane protein complex” (GO:0098797, corrected *p*-value =2.36E-08) and “cell cycle process” (GO:0022402, corrected *p*-value =5.04E-07) (Fig. 5c, Table S4). KEGG analysis indicated that these genes are closely related to RNA transport (Fig. 5g). In addition, 13 hub genes were identified (Fig. 5i, Table S8), including *LOC109939959* encoding a homolog of the *Arabidopsis thaliana* DNA demethylase REPRESSOR OF SILENCING 1 (*ROS1*, *AT2G36490*). The Arabidopsis *ros1* mutant shows higher methylation levels of many gene promoters and increased susceptibility to pathogens [33–36]. Another hub gene, *LOC103631592*, might also be involved in regulating methylation levels, because its Arabidopsis homolog *AT5G04290* (*SPT5L*) is required for RNA-directed DNA methylation [37, 38]. Consistently, GO analysis also indicated that genes in the green module were enriched in “demethylation” (GO:0070988, corrected *p*-value = 0.0019), “gene silencing by RNA” (GO:0031047, corrected *p*-value = 0.0184), and “gene silencing” (GO:0016458, corrected *p*-value = 0.0192). Collectively, we speculated that the change of the genome methylation level and gene silencing might be related to the maize kernel resistance in the early stage of *A. flavus* infection.

In the yellow module, the expression level of genes was drastically reduced at the T2 stage in the S and SC groups, and the decrease in S2 was more pronounced than that in SC2 (Fig. 5e). GO enrichment analysis revealed that 12 GO terms were significantly over-represented (corrected *p*-value < 0.05), including “small molecule metabolic process” (GO:0044281, corrected *p*-value = 1.94E-10) and “cellular response to endogenous stimulus” (GO:0071495, corrected *p*-value = 0.0450) (Fig. 5f, Table S5). The top two pathways with the highest significance were “biosynthesis of antibiotics” and “fatty acid biosynthesis” based on KEGG pathway analysis (Fig. 5h). Additionally, a total of 23 hub genes in this module were screened out. Five (*fab1*, *LOC100192829*, *LOC100281497*, *gpm519*, and *fae1*) and two (*pco065117* and *LOC100191601*) of these hub genes are directly related to fatty acid biosynthesis and lipid metabolic processes, respectively (Fig. 5j, Table S8).

In the brown module, the expression of these genes increased in the SC and S groups, and the increase in S2 was more evident (Fig. 6b). According to GO and KEGG analysis, only six terms (corrected *p*-value< 0.05) and three enriched pathways (*Q* value< 0.05) were identified (Fig. 6c, g, Table S6). Of the 19 hub genes identified in this module (Fig. 6i, Table S8), three Barwin family proteins (*LOC100282340*, *LOC103652813*, and *LOC103652814*) with chitinase activity might play vital roles as PR proteins in restriction of *A. flavus* infection [39, 40]. *LOC100193776*, as the ortholog of *LAZY1* in rice, participates in regulating auxin transport and auxin signaling, was also a hub gene of the brown module [41]. In addition, four other hub genes (*LOC100382449*, *pco080661a*, *LOC100273283*, and *LOC103632841*) are considered to be involved in the response to various biotic or abiotic stresses.

In the blue module, the expression of genes increased in the RC and R groups, and the trends were more obvious after *A. flavus* infection (R) (Fig. 6e). According to GO and KEGG analysis, there were 71 terms and 32 pathways with statistical significance (Fig. 6f, h, Table S7). Remarkably, in the blue module, 226 genes were involved in “oxidation-reduction process” (GO:0055114, corrected *p*-value = 0.0017) and 8 genes were associated with “plant-type hypersensitive response” (GO:0009626, corrected *p*-value = 0.0075) (Fig. 6f, h). KEGG analyses also showed that genes in this module were enriched in pathways such as “biosynthesis of antibiotics”, “biosynthesis of amino acids”, “glycolysis/gluconeogenesis”, and “fatty acid degradation” (Fig. 6g). Totally 43 hub genes in this module were identified in this module (Fig. 6j, Table S8). Six (*LOC103636223*, *LOC100382469*, *LOC103650090*, *LOC100284999*, *LOC103626647*, and *pco102102*) of them are closely related to the biosynthesis of antibiotics. Another hub gene, *IDP2565* encoding aPR10 protein, is vital in maize host defense, and *PR10* RNAi-silenced mature kernels showed more fungal colonization and aflatoxin production [17, 42]. The hub gene *LOC100274556* is a homolog of *AT1G68010*, whose protein possesses catalase activity and confers tolerance to multiple abiotic stresses in Arabidopsis [43, 44].

Taken together, a total of 110 hub genes in the 7 modules correlated with different infection stages. These genes were enriched in the GO terms, “cellular amine metabolic process”, “defense response to fungus”, and “fatty acid synthase activity” (Figure S12, Table S9). The top two KEGG pathways were “fatty acid biosynthesis” and “biosynthesis of antibiotics” (Figure S12). Hence, these hub genes and biological pathways might play an important role in modulating the defense response to *A. flavus* infection in maize.

### Integration of previously identified candidate genes and transcriptome data

**Fig. 7:**
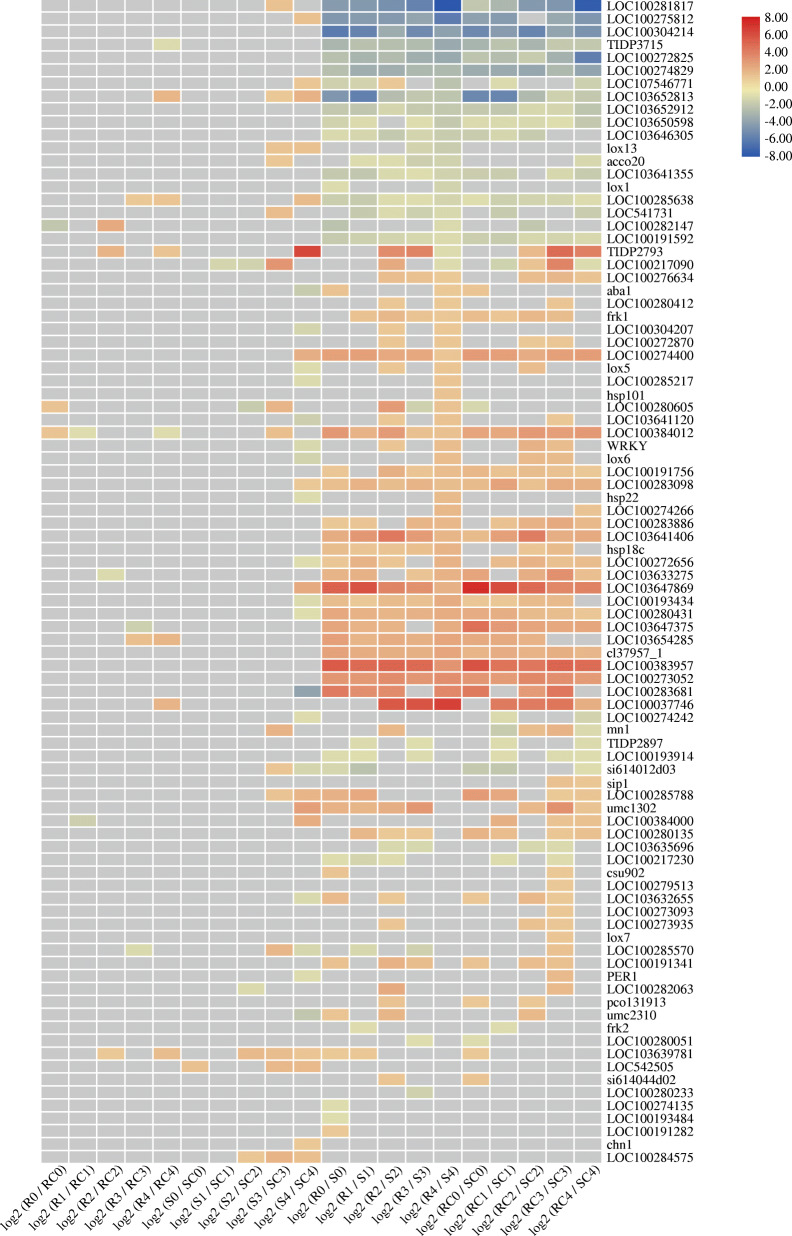
Heat map of 90 candidate genes for maize resistance to *A. flavus* infection and/or aflatoxin contamination. Red indicates up-regulated, blue indicates down-regulated. The gray cells indicate no differential expression. The color scale represents Z-score

A list of 195 candidate genes for maize resistance to *A. flavus* infection and/or aflatoxin contamination has been reported [45]. Interestingly, we noticed differential expression of these genes in both lines in the early stage of infection. In the first 6 hpi, 90 of these genes were found to be differentially expressed (Fig. 7). The expression differences between genotypes (RC vs. SC, R vs. S) were significantly higher than those between treatments (RC vs. R, SC vs. S). Compared with the control groups (RC and SC), 14 and 42 genes were differentially expressed in AF99 and AF32, respectively. Eight of these genes (*LOC103652813*, *LOC103639781*, *LOC100280605*, *TIDP2793*, *LOC100285638*, *LOC100384012*, *LOC100384000*, and *LOC103633275*) were induced in both lines. At the T4 stage, 6 genes were up-regulated in AF99, including Barwin-like (*LOC103652813*), the transcription factor MYB41 (*LOC100037746*), chitinase 2 (*LOC100285638*), WRKY transcription factor 23 (*LOC103654285*), probable WRKY transcription factor 53 (*LOC103639781*), and *LOC100272820* encoding an unknown protein. Among them, *LOC103652813*, *LOC100285638*, *LOC100272820*, and *LOC103639781* were also up-regulated in AF32. In addition, 18 genes were down-regulated in AF32, while only 2 genes were down-regulated in AF99, indicating that more disease-resistance were induced and then contributed to the resistance of AF99.

We also analyzed the relationship between these candidate genes and our hub genes obtained by WGCNA, and found that 3 of the 110 hub genes were in the list. Two hub genes in the blue module, *LOC100279513* and *LOC100285591*, were either linked to QTLs in two mapping populations or associated with aflatoxin accumulation in the previously reported GWAS panel [45]. One hub gene (*LOC103652813*) of the green module, located in bin 4.02, was also in the consolidated list [45].

## Discussion

The molecular mechanisms and precise regulatory network underlying the defense system of maize kernel in response to *A. flavus* in the early stage are limited. In this work, we focused on the transcriptome reprogramming of resistant and susceptible lines within 6 h of fungal infection. DEGs induced by *A. flavus* infection or between the two lines were analyzed. More than 300 DEGs were identified in both lines at the T0 stage (Table 1), while only one genes were found at the early infection stage in a previous study [29], which reveals that the response of maize kernels to fungal invasion is very rapid. This difference might be due to the improved fungi inoculation method employed in our study. Here, maize kernels were cut longitudinally and immersed into the spore suspension. The full contact between maize cells and *A. flavus* may eliminate the interference caused by the seed coat. The kernels inoculated at 0, 0.5, 1.5, 3, 6 hpi were sampled in this study and DEGs varied at each time point, which means a dynamic regulation occur in the early stage. The density of our sampling time will facilitate our understanding of the precise expression regulation network of maize against *A. flavus* in the initial stages*.* Compared with mock-inoculated groups, the susceptible line AF32 had more DEGs than the resistant line AF99, except for at the T0 stage (Table 1). This finding is in agreement with the previous results that 214 and 2159 genes were induced in resistant and susceptible kernels at 72 hpi, respectively [46]. These results suggest that the susceptible lines are vulnerable to *A. flavus* infection, leading to a dramatic change in gene expression after inoculation.

Based on the dynamic transcriptome analysis, *A. flavus* infection induced a large number of DEGs in both lines, most of which are related to host defense (Fig. 2). Among them, 84% of DEGs were up-regulated after inoculation in AF99(R), while only 48% were up-regulated in AF32. (Fig. 2). Besides, a majority of 90 candidate resistance genes showed a higher basic expression level in AF99 than in AF32 (Fig. 7). Taken together, these might be the leading cause of the difference in resistance (Fig. 2).

Integrated GO analysis, KEGG analysis and WGCNA demonstrated that *A. flavus* resistance in maize is genotype−/ stage-specific (Figs. 2, 3, 4, 5, 6). WGCNA is a well-established tool in systems biology, which can be used to describe correlation patterns among genes across multiple samples. Through WGCNA, we adopted the whole-genome expression data to construct complete co-expression networks, and 7 modules were considered to be correlated with different infection stages (Fig. 4). GO and KEGG analysis in these modules revealed that the genes participated in plasma membrane protein complex, cell cycle process, RNA transport, and DNA methylation were affected in the initial stage of the infection (T0 and T1); some secondary metabolic processes, like biosynthesis of antibiotics, fatty acid biosynthesis, and biotin metabolites, were activated in response to infection at the T2 stage; more DEGs or hub genes involved in hormone signaling, antibiotic and fatty acid biosynthesis, lipid storage, and hypersensitive cell death responses (HRs) were discovered at stage T3 and T4 (Figs. 3, 4, 5, 6, S6, S8). Therefore, our WGCNA provided new genetic resources for molecular breeding and deepened our understanding of maize resistance to *A. flavus*.

*A. flavus* is a necrotrophic fungal pathogen that feeds on dead host cells. Traditionally, it is believed that the HR, as well as HR-triggered programmed cell death (PCD) can enhance necrotrophic pathogen virulence [47–49]. However, in recent years, some studies have reported that increasing the expression of some genes that trigger the HR may also enhance plant resistance to necrotrophic pathogens [50, 51]. Herein, functional analysis of hub genes and GO enrichment analysis of the 7 modules correlated with infection stages revealed that genes involved in HR and PCD might play a crucial role in maize resistance against *A. flavus* (Figs. 5, 6, 7, Table S8). We analyzed 35 genes related to the HR and PCD and found 11 were in the blue module and up-regulated in AF99(R) at the T4 stage (Figure S13). Among them, *LOC100502274* is a homolog of *AT4G38360* (*LAZ1*) who contributes to PCD associated with the HR in Arabidopsis [52, 53]. However, 4 of the 35 genes were up-regulated at the T4 stage in AF32(S), and 3 of which were clustered in the brown module (Figure S13). In particular, two genes (*LOC103652814* and *pco080661a*) are the hub genes of the brown module, implying that HRs were stimulated at the T4 stage in the susceptible line. These results indicated that PCD and HRs might be triggered after *A. flavus* inoculation in both lines, and further experiments are needed to verify their relationship with maize host defense against *A. flavus*.

The KEGG enrichment analyses suggested that genes related to antibiotics synthesis might be involved in regulating host defense (Figs. 3, 5, 6, S8, S12). Plant-derived antibiotics are antimicrobial secondary metabolites produced by the plant. They contain both preformed antifungal compounds constitutively present in healthy plants and induced antifungal compounds (phytotoxin) synthesized in response to pathogen invasion [54]. In maize, dynamic regulation of several antibiotics, including benzoxazinoids, phenylpropanoids, oxylipins, and terpenoids, contributes greatly to host resistance when challenged with diverse pathogens [55–61]. In our study, genes regulating phenylpropanoid, diterpenoid, and terpenoid backbone biosynthesis were up-regulated in response to fungus attack in both the resistant and susceptible lines (Figure S6). Based on the results of WGCNA, 58 and 388 genes involved in biosynthesis of antibiotics were enhanced in AF99 in the yellow and blue modules, respectively; while only 38 related genes were found in AF32 in the brown module. These results suggested that AF99 might have a greater ability to synthesize antibiotics (Figs. 5, 6). Therefore, we speculated that plant-derived antibiotics participate in the regulation of maize resistance to *A. flavus* in the early stage of infection and might be an important factor underlying resistance variation among genotypes.

## Conclusions

Collectively, RNA-seq data generated from two maize lines with contrasting resistance in the pre-harvest and post-harvest inoculation provided a robust resource to study maize kernel resistance to *A. flavus.* Herein, we investigated the genotype-specific and infection stage-specific response of maize against *A. flavus* infection, and found that inhibition of some defense pathways might lead to the reduced host resistance in the susceptible line. We also constructed gene co-expression networks during the first 6 hpi by WGCNA, and discovered that DNA methylation, biosynthesis of antibiotics, fatty acid biosynthesis, hormone signaling, and HRs greatly influenced host resistance. Further, the 110 hub genes identified in this work could be important targets for maize resistance against *A. flavus* in future breeding efforts.

## Methods

### Plant materials and growth conditions

Two maize inbred lines, AF99 (resistant line) and AF32 (susceptible line), were selected from recombinant inbred lines derived from a cross between RA and Z58. The parental line RA was obtained from a cross between two Chinese elite inbred lines, Ye478 and Dan340, which showed excellent resistance to *Aspergillus flavus* in years of repeated experiments [62]. The other parental line, Z58, is a Chinese elite inbred line.

All the individuals were planted in the fields at the Experimental Station of Yangzhou University (18°18′06″N 109°39′32″E) during the 2019 growing season. The plants were grown in 0.55 × 0.25 m plots, and each plant was self-pollinated to ensure enough materials for further investigation.

### Inoculation method and phenotypic evaluation

The *A. flavus* strain used in this study was isolated by Professor Yin Shixue (College of Environmental Science and Engineering of Yangzhou University) [63]. *A. flavus* was grown on modified Czapek agar medium at 30 °C under dark cultivation for 7 days, and a conidial suspension (2 × 10^6^ spores mL^− 1^) was prepared with sterile water before inoculation.

For the pre-harvest inoculation, maize ears at 15 days after pollination were selected and artificially inoculated with the nail punch method. Two injections per ear were performed with 1 ml of spores per injection. The disease resistance phenotype was identified at harvest by measuring the extent of *A. flavus* growth on the ears.

For the post-harvest inoculation, the harvested ears were inoculated with a conidial suspension in the laboratory. The inoculation procedure and the scoring for kernel resistance to *A. flavus* infection (RAI) were conducted according to the procedure described in our previous study [11, 64]. The RAI scores were divided into 11 grades according to the proportion of hyphae and spores covering the maize kernel surface (0 for no infection and 10 for complete infection), and each level represents 10% coverage.

### RNA-seq and DEG analysis

Well-developed maize ears of AF99 and AF32 were selected at 15 d after pollination and kernels in the middle of ears were collected for further study. Each kernel was cut longitudinally with a sterile scalpel and divided into two equal parts; half of the kernels were immersed in a spore suspension for 5 min, and the other half were immersed in sterile water for 5 min, recorded as 0 h. Then, all kernel halves were transferred to modified Czapek agar medium and cultured in an incubator at 30 °C. At least four kernel halves were collected as one sample at 0, 0.5, 1.5, 3, and 6 h (named T0, T1, T2, T3, and T4, respectively), and rapidly cooled with liquid nitrogen and stored in a deep freezer at − 80 °C. Each sample had three biological repetitions to minimize experimental error. The detailed treatment method was described previously [31].

The total RNA was isolated with the ethanol precipitation protocol and CTAB-PBIOZOL reagent (Vazyme Biotech). RNA was qualified and quantified using a NanoDrop spectrophotometer and an Agilent 2100 bioanalyzer (Thermo Fisher Scientific, MA, USA). Transcriptome sequencing was performed on the BGISEQ500 platform (BGI-Shenzhen, China). The raw reads containing the sequencing adapter or with over 5% unknown bases (‘N’ base) and those whose low-quality reads base ratio (base quality ≤5) was greater than 20% were filtered by SOAPnuke (v1.5.2) [65]. Then the HISAT2 (v2.0.4) [66] was applied to align the clean reads to the maize reference genome GCF_000005005.2_B73_RefGen_v4(https://www.ncbi.nlm.nih.gov/assembly/GCF_000005005.2).

RSEM (v1.2.12) [67] was used to calculate the fragments per kilobase of transcript per million mapped reads (FPKM). DESeq2(v1.4.5) [68] was used to identify the DEGs with the criterion of fold change ≥2.00 and adjusted *p*-value ≤0.05.

### Real-time RT-PCR

cDNA was synthesized from 0.5 μg of total RNA with HiScript®Q RT SuperMix for qPCR (Vazyme, China) according to the protocol. Quantitative RT-PCR was conducted using the StepOnePlus Real Time PCR system with ChamQ SYBR qPCR Master Mix (Vazyme, China). Gene specific primers (Table S2) were designed with QuantPrime qPCR primer design tool (https://quantprime.mpimp-golm.mpg.de). The primers ZmUBQ-qRT+/− were used to amplify the ubiquitin1 as control, and the relative gene expression data was calculated using 2^-△△Ct^ method. Each sample had three biological repetitions with three technical replicates to minimize experimental error.

### Gene ontology and KEGG pathway enrichment analysis

ClueGO (v. 2.5.7) [69], a Cytoscape plug-in software of Cytoscape, was employed to conduct the gene ontology (GO) enrichment analysis (*p*-value corrected with Bonferroni step down ≤0.05). KEGG (https://www.kegg.jp/) enrichment analysis was performed by Phyper (https://en.wikipedia.org/wiki/Hypergeometric_distribution) based on the hypergeometric test (*p*-value corrected with Bonferroni step down ≤0.05). MAPMAN software [70] was used to identify the genes in AF99 and AF32 that responded to *A. flavus* infection.

### Weighted gene co-expression network analysis

The R package for weighted gene co-expression network analysis (WGCNA) was used for describing correlation patterns among genes across multiple samples [71, 72]. All gene expression data were standardized based on log_2_(1 + FPKM) values, and soft threshold = 12, based on the scale-free topology criterion, was selected to generate an adjacent matrix. Then the adjacency matrix was converted to a topological overlap matrix (TOM), and the genes were hierarchically clustered based on dissimilarity between genes. The dynamic tree-cutting algorithm was used to cut the hierarchal clustering dendrogram (mergeCutHeight = 0.25) and modules were defined (the minimum number of modules was 30). To estimate the association of modules with gene-specific expression (genotype-specific or infection stage-specific expression), the binary indicator (stages/genotypes = 1 and all other samples = 0) was used as described [72, 73]. At each time point, stage−/genotype-specific modules (|r| > 0.35, *p* < 0.05) and hub genes (|gene significance| > 0.4 and |intramodular connectivity in interesting modules| > 0.9) were identified. Cytoscape software version 3.6.0 [74] was used to visualize co-expression networks. The Venn diagrams and heatmaps were drawn using the TBtools [75].

## Supplementary Information

**Additional file 1: Figure S1.** Pair-wise Pearson’s correlation coefficients of the sequencing data of 60 samples. **Figure S2.** Dynamic expression patterns of genes during infection by real-time RT-PCR. **Figure S3.** Venn diagrams of specific and common DEGs responding to *A. flavus* in AF99 and AF32 (infection vs. mock-treatment) at different time points (a, 0 hpi; b, 0.5 hpi; c,1.5 hpi; d, 3 hpi; e, 6 hpi). **Figure S4.** Venn diagram of up-regulated and down-regulated DEGs at different time points in AF99. **Figure S5.** Venn diagram of up-regulated and down-regulated DEGs at different time points in AF32. **Figure S6.** KEGG pathways enriched in down- and up-regulated genes responding to *A. flavus* in AF99 and AF32 (infection vs. mock-treatment) at different time points. Color depth represents the corrected *p*-value. **Figure S7.** MapMan-based visualization of an overview of the cellular response at the T4 stage in AF99(a) and AF32(b).

**Additional file 2: Figure S8.** KEGG analysis of the specific differentially expressed genes of fungal-inoculated kernels between AF99 and AF32. Color depth represents the degree of significance, as shown in the scale at the right (corrected p-value). **Figure S9.** Determination of soft-thresholding power (β). a: scale-free topology fit index as a function of the soft-thresholding power, the red line indicates that R^2^ is equal to 0.85. b: mean connectivity as a function of the soft-thresholding power. **Figure S10.** The sample dendrogram and trait heatmap. Lines: “0” for AF32 and “1” for AF99; Ino: “0” for mock and “1” for *A. flavus* inoculation; Time: “0, 0.5, 1.5, 3, 6” indicate the time after inoculation; RC0: “1” for the three biological repetitions of AF99 (mock-treated) at the T0 stage and “0” for other samples; R0: “1” for the three biological repetitions of AF99 (*A. flavus* inoculation)) at the T0 stage and “1” for other samples; SC0:“1” for the three biological repetitions of AF32 (mock-treated) at the T0 stage and “0” for all the other samples; S0:“1” for the three biological repetitions of AF32 (*A. flavus* inoculation) at the T0 stage and “0” for all the other samples; the rest are similar as “RC0”, “R0”, “SC0” and “S0”. **Figure S11.** Scatterplots of gene significance versus module membership for the brown/R0(a), magenta/SC1(b), pink/S1(c), black/S2(d) and brown/S3(e), with correlations and *p*-values indicated. **Figure S12.** GO and KEGG pathway analysis of 110 hub genes identified in this study. a. GO analysis of 110 hub genes. Each circle represents an enrichment category, and the size of the circle indicates the number of genes. Detailed enrichment results are shown in Table S9 b. The top 20 associated KEGG pathways for the 110 hub genes. **Figure S13.** Heat map of 35 genes associated with the hypersensitive response and programmed cell death. Red indicates high expression, blue indicates low expression. Remove very low expression genes (average FPKM< 0.5).

**Additional file 3: Table S1.** Detail information of sequencing reads from different sample groups.

**Additional file 4: Table S2.** List of primers used for Real-time RT-PCR.

**Additional file 5: Table S3.** Information of all genes in MapMan.

**Additional file 6: Table S4.** GO analysis of genes in the green module.

**Additional file 7: Table S5.** GO analysis of genes in the yellow module.

**Additional file 8: Table S6.** GO analysis of genes in the brown module.

**Additional file 9: Table S7.** GO analysis of genes in the blue module.

**Additional file 10: Table S8.** List of hub genes identified in this study.

**Additional file 11: Table S9.** GO analysis of the 110 hub genes.

## Data Availability

The raw data are available from the National Center for Biotechnology Information SRA Explorer (https://sra-explorer.info/?) under accession PRJNA691427.

## Abbreviations

*A. flavus*: *Aspergillus flavus*
hpi: Hours post inoculation
DEG: Differentially expressed genes
WGCNA: Weighted gene co-expression network analysis
QTL: Quantitative trait locus
GWAS: Genome-wide association studies
PCD: Programmed cell death
HR: Hypersensitive cell death response
MAD: Median absolute deviation
