# Supplementary material for: Comparative transcriptome profiling and co-expression network analysis uncover the key genes associated withearly-stage resistance to Aspergillus flavus in maize
Source: BMC Plant Biol. 2021 May 13;21:216. doi: 10.1186/s12870-021-02983-x (PMC8117602; doi:10.1186/s12870-021-02983-x)
Supplement: Supplementary file 1 — Additional file 1: Figure S1. Pair-wise Pearson’s correlation coefficients of the sequencing data of 60 samples. Figure S2. Dynamic expression patterns of genes during infection by real-time RT-PCR. Figure S3. Venn diagrams of specific and common DEGs responding to A. flavus in AF99 and AF32 (infection vs. mock-treatment) at different time points (a, 0 hpi; b, 0.5 hpi; c,1.5 hpi; d, 3 hpi; e, 6 hpi). Figure S4. Venn diagram of up-regulated and down-regulated DEGs at different time points in AF99. Figure S5. Venn diagram of up-regulated and down-regulated DEGs at different time points in AF32. Figure S6. KEGG pathways enriched in down- and up-regulated genes responding to A. flavus in AF99 and AF32 (infection vs. mock-treatment) at different time points. Color depth represents the corrected p-value. Figure S7. MapMan-based visualization of an overview of the cellular response at the T4 stage in AF99(a) and AF32(b). [file 12870_2021_2983_MOESM1_ESM.pdf]

Comparative transcriptome profiling and co-expression network analysis uncover the key genes associated with early-stage resistance to *Aspergillus flavus* in maize

Huanhuan Liu[$1], Haofeng Wu[$1], Yan Wang[1], Huan Wang[1], Saihua Chen[1*], Zhitong Yin[1*]

1, Jiangsu Key Laboratory of Crop Genetics and Physiology/Co-Innovation Center for Modern Production Technology of Grain Crops/Key Laboratory of Plant Functional Genomics of the Ministry of Education/Joint International Research Laboratory of Agriculture & Agri-Product Safety of the Ministry of Education, Yangzhou University, Yangzhou, 225009, China.

＄These authors contributed equally

*Corresponding author:

Zhitong Yin（ztyin@yzu.edu.cn）

Saihua Chen（chensaihua@yzu.edu.cn）

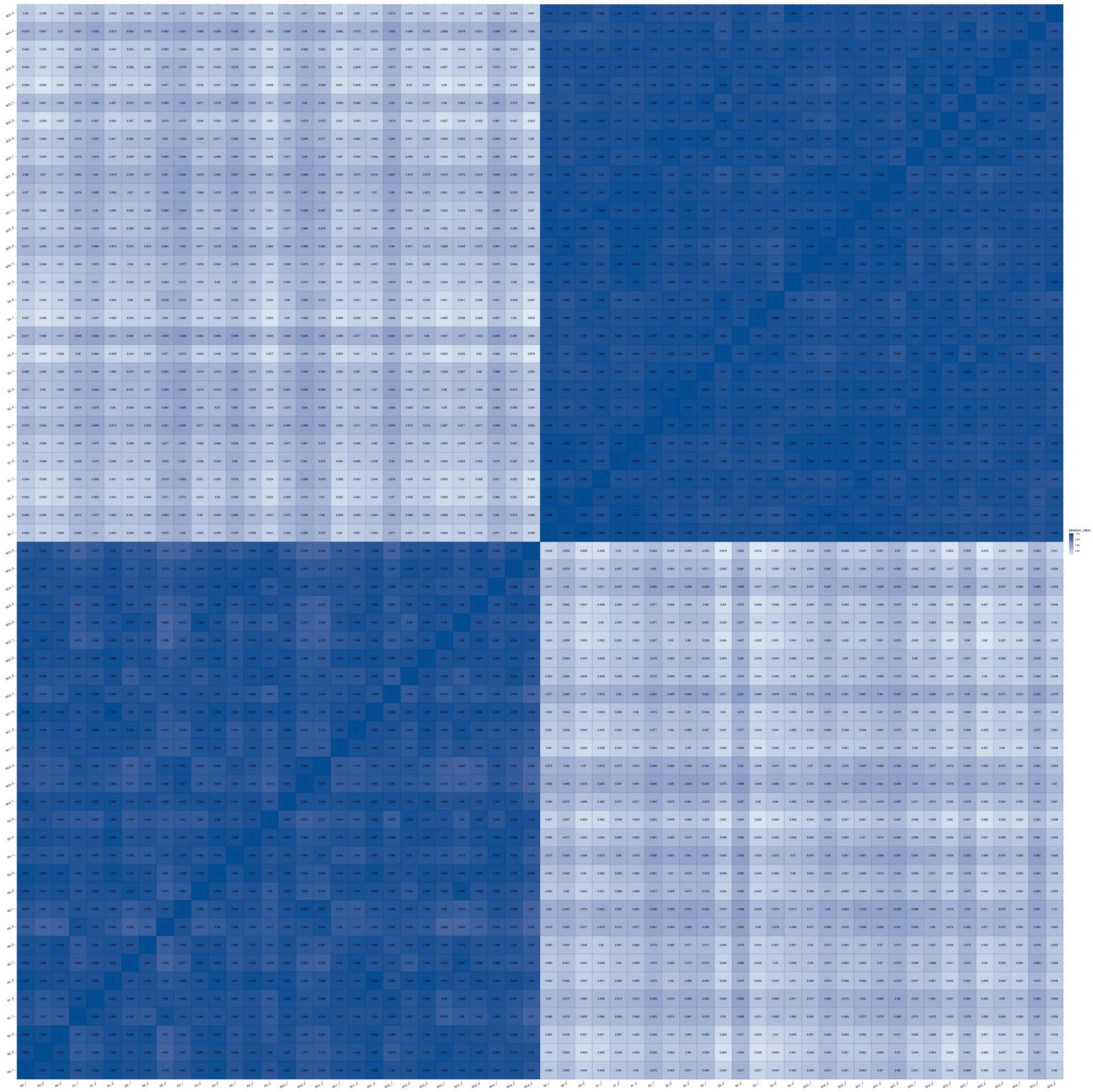

Figure S1. Pair-wise Pearson’s correlation coefficients of the sequencing data of 60 samples.

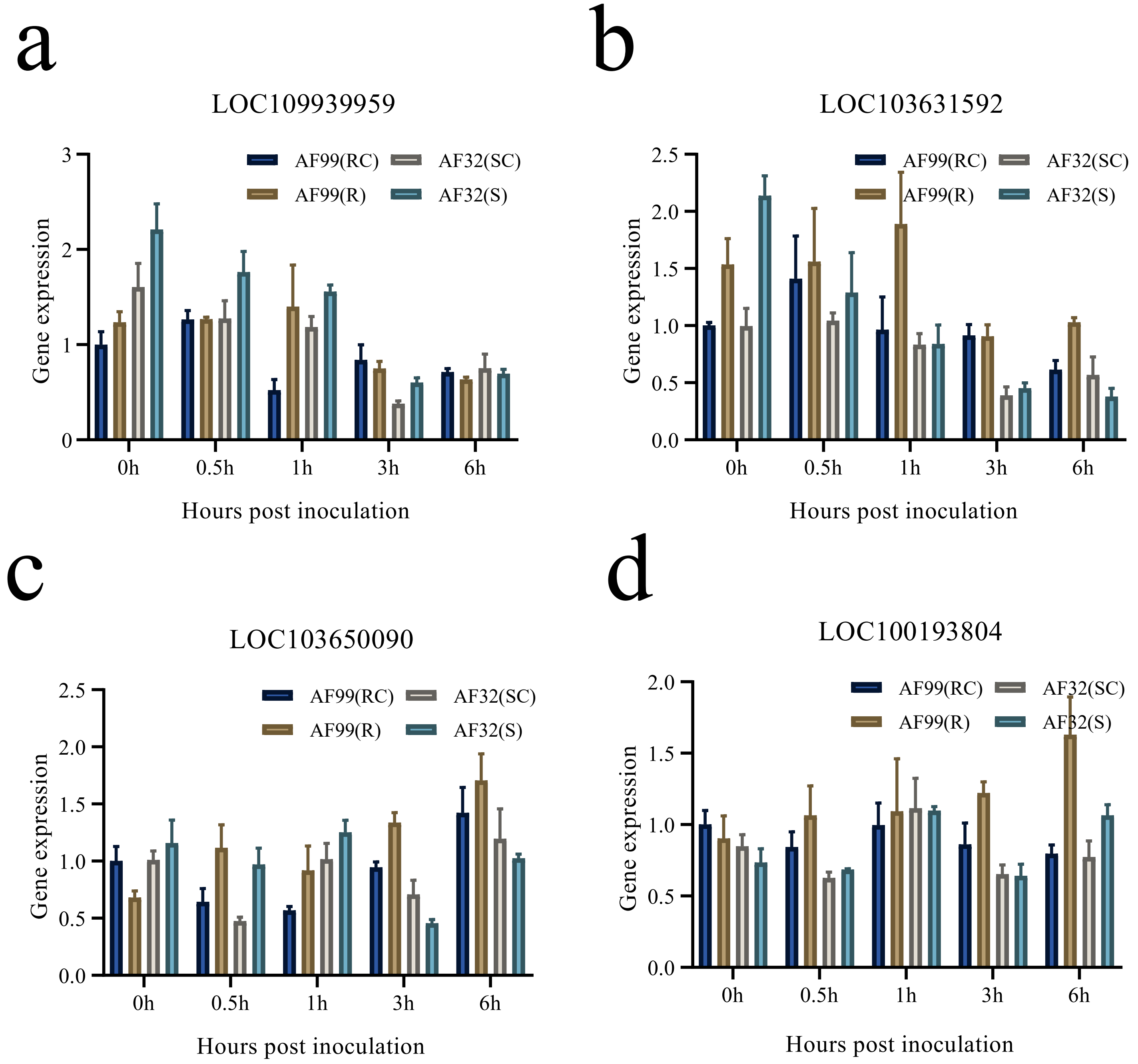

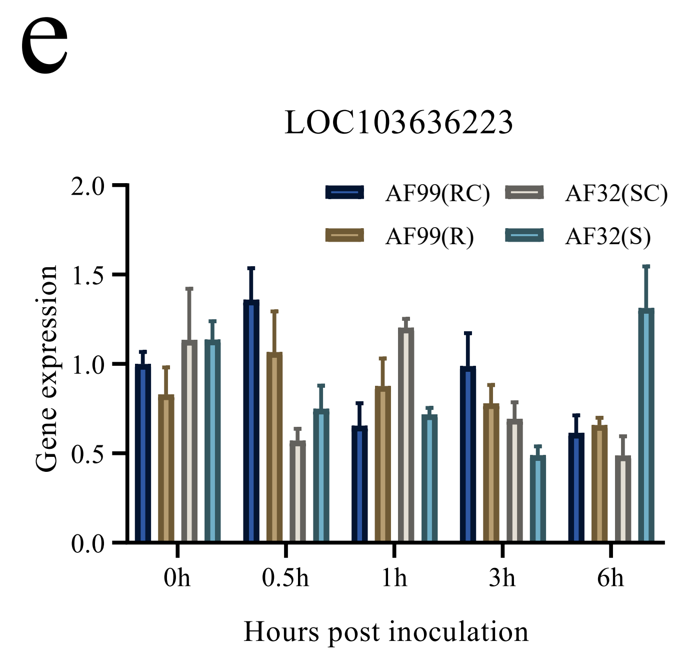

Figure S2. Dynamic expression patterns of genes during infection by real-time RT-PCR.

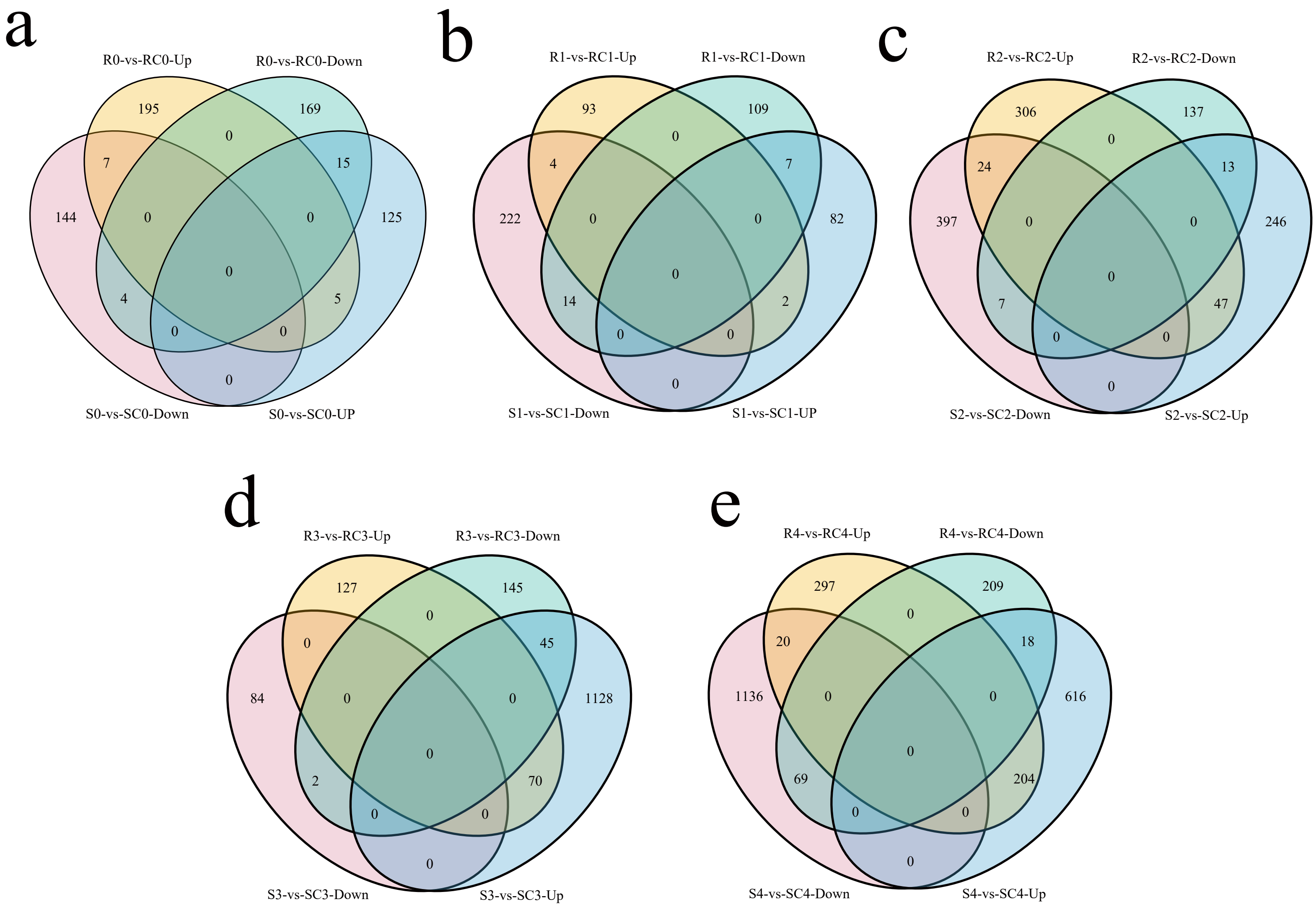

Figure S3. Venn diagrams of specific and common DEGs responding to *A. flavus* in AF99 and AF32 (infection vs. mock-treatment) at different time points (a, 0 hpi; b, 0.5 hpi; c,1.5 hpi; d, 3 hpi; e, 6 hpi).

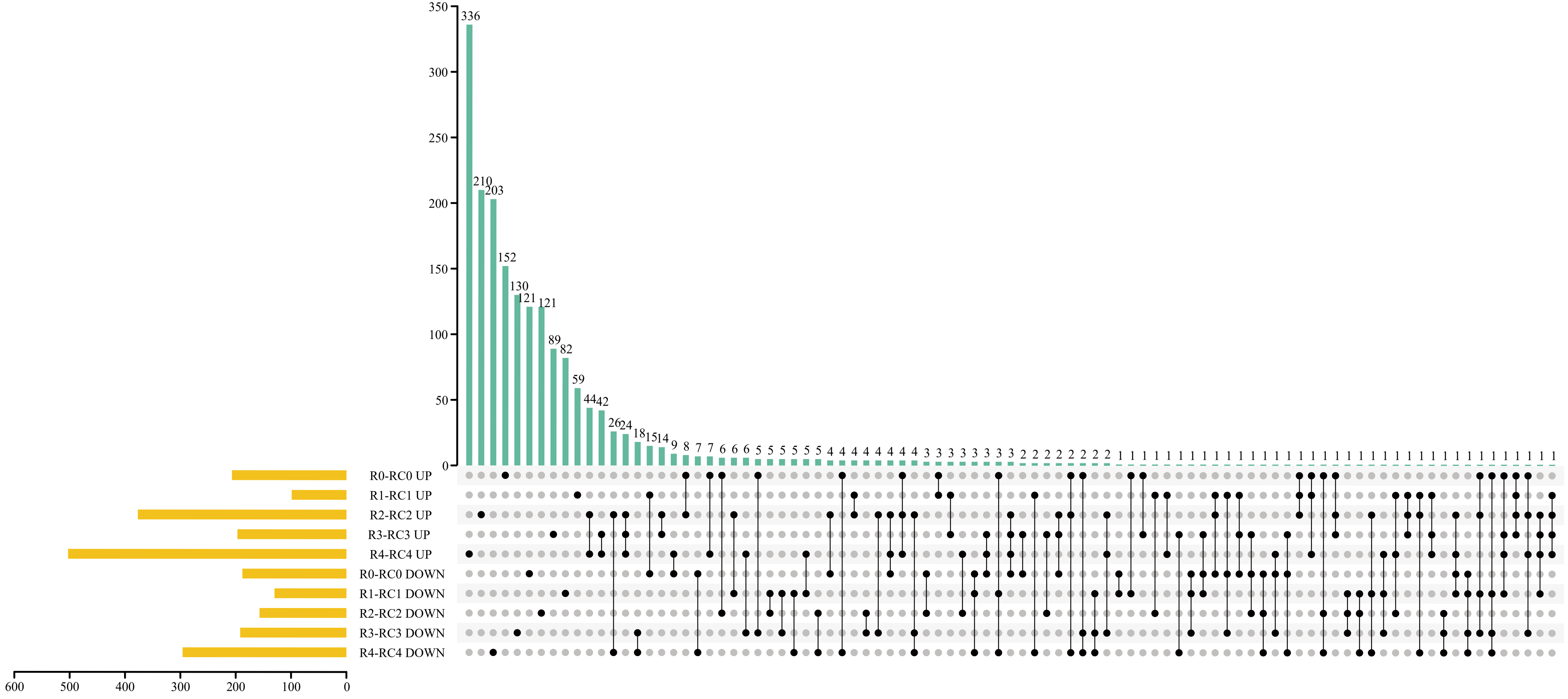

Figure S4. Venn diagram of up-regulated and down-regulated DEGs at different time points in AF99.

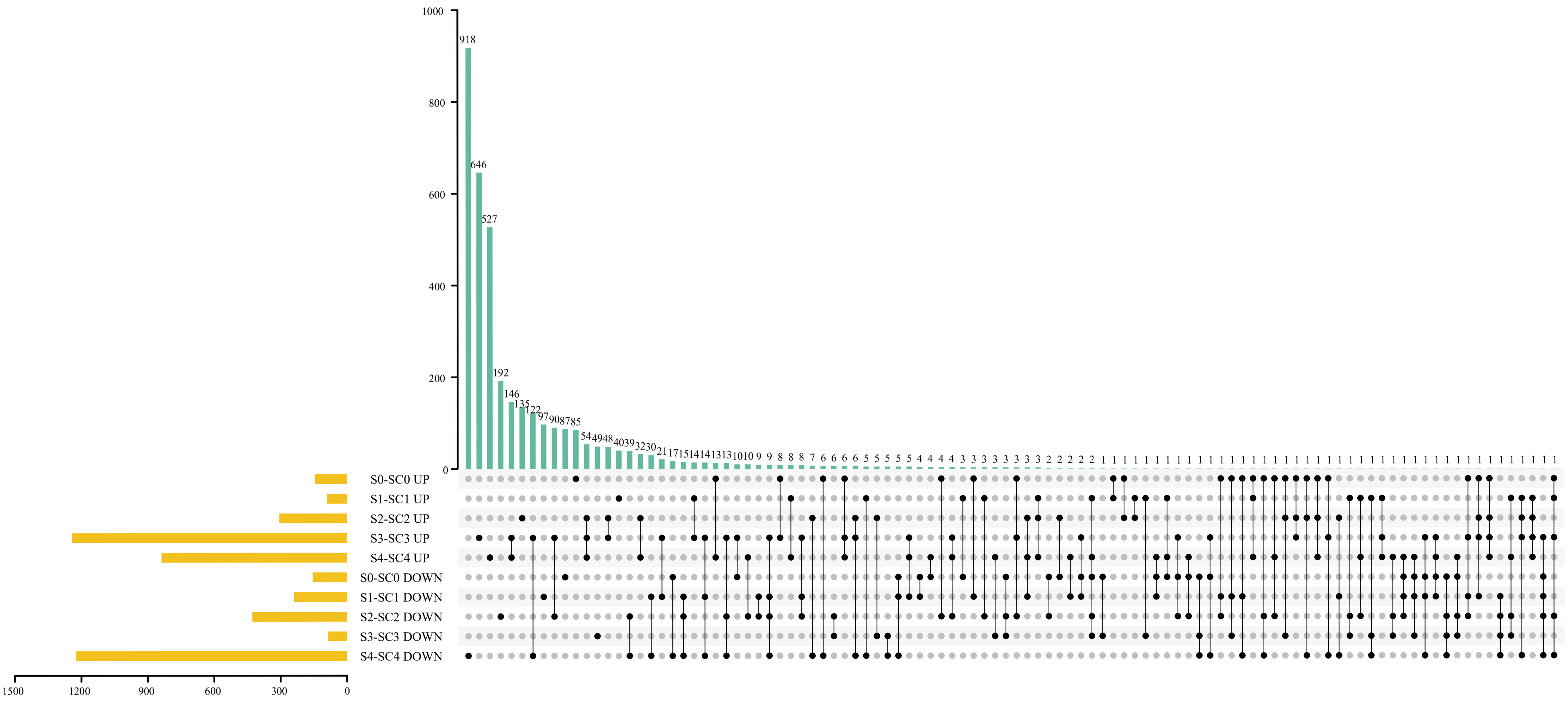

Figure S5. Venn diagram of up-regulated and down-regulated DEGs at different time points in AF32.

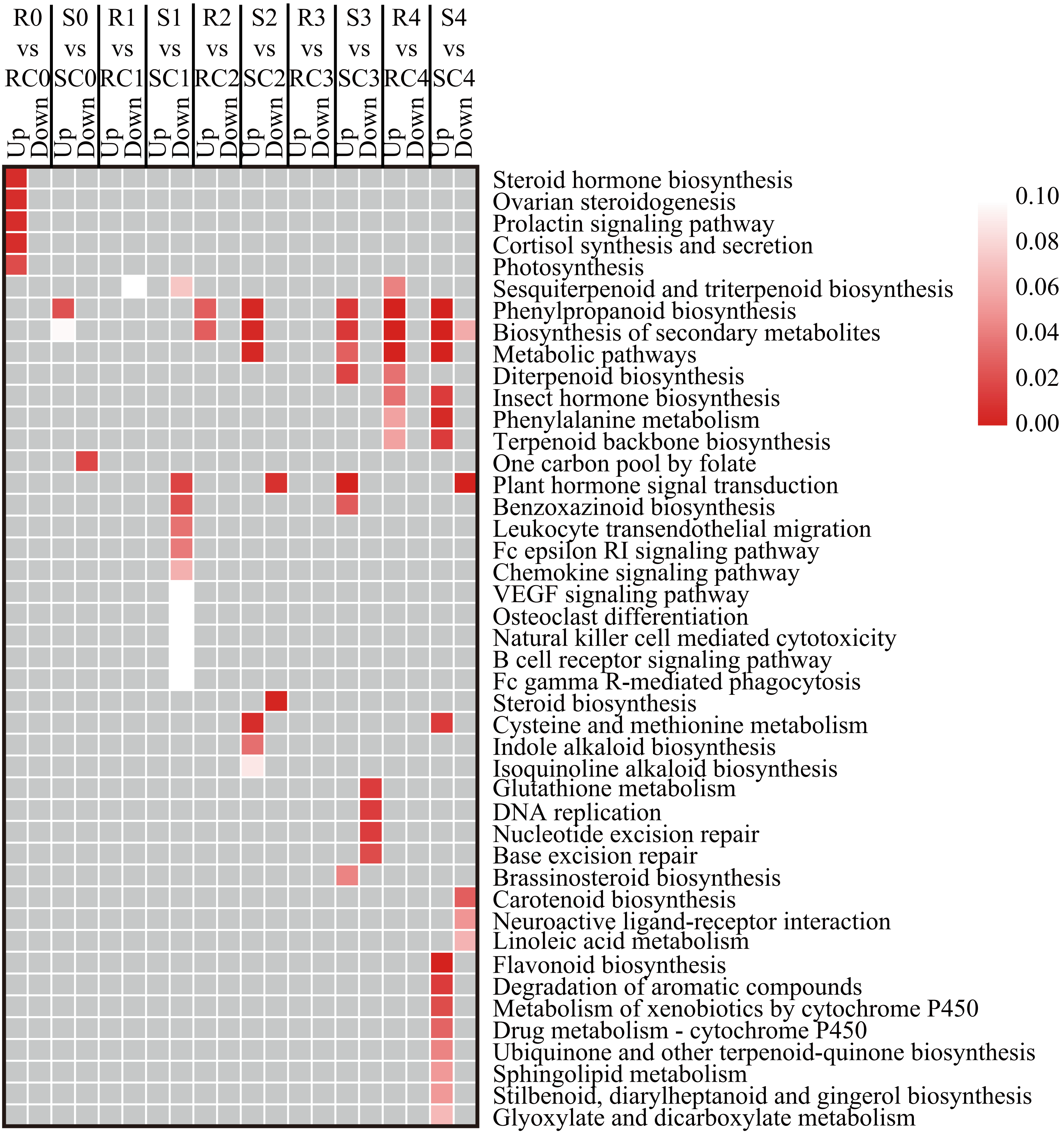

Figure S6. KEGG pathways enriched in down- and up-regulated genes responding to *A. flavus* in AF99 and AF32 (infection vs. mock-treatment) at different time points. Color depth represents the corrected p-value.

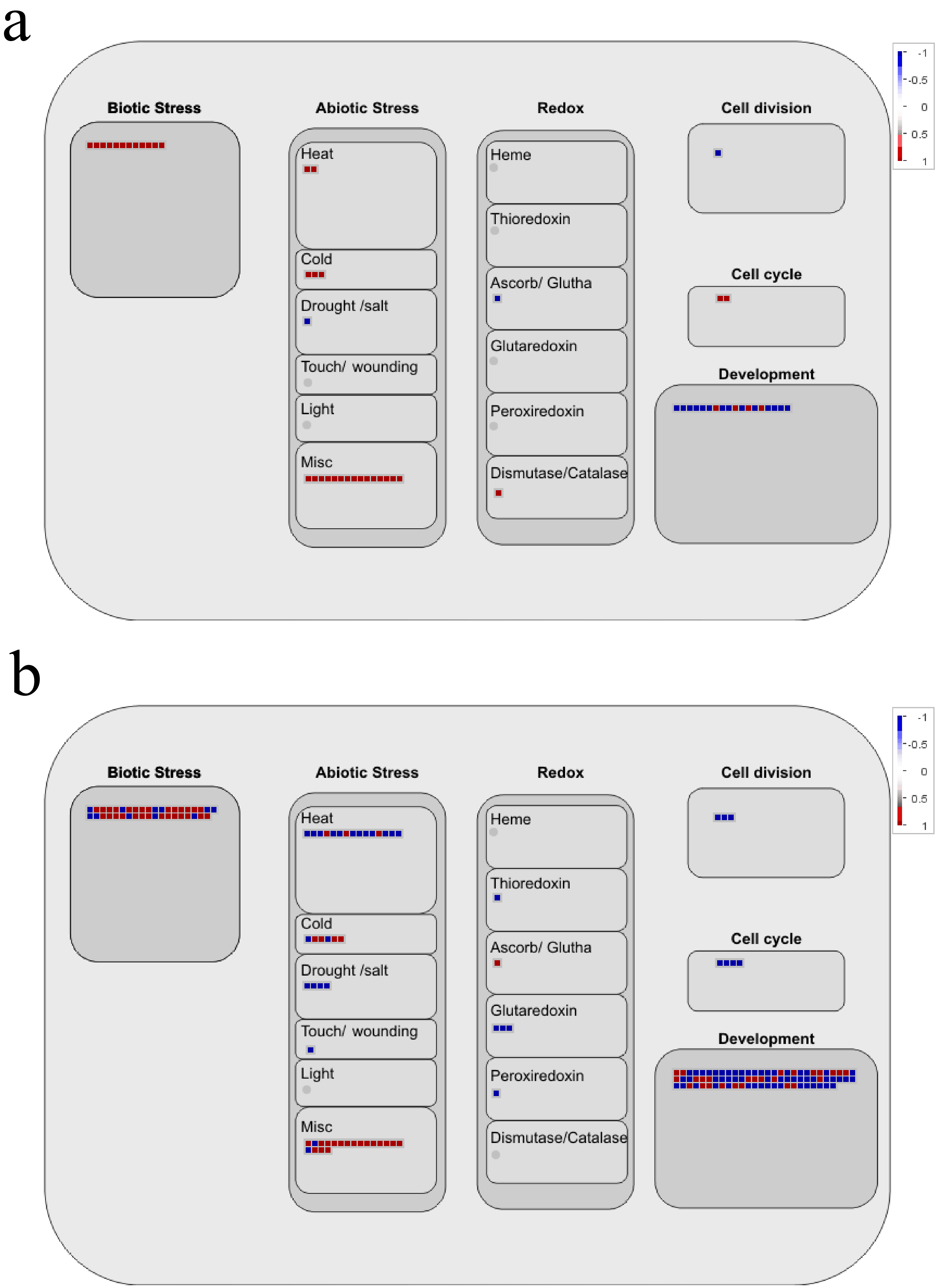

Figure S7. MapMan-based visualization of an overview of the cellular response at the T4 stage in AF99(a) and AF32(b).
