# Supplementary material for: Comparative transcriptome profiling and co-expression network analysis uncover the key genes associated withearly-stage resistance to Aspergillus flavus in maize
Source: BMC Plant Biol. 2021 May 13;21:216. doi: 10.1186/s12870-021-02983-x (PMC8117602; doi:10.1186/s12870-021-02983-x)
Supplement: Supplementary file 2 — Additional file 2: Figure S8. KEGG analysis of the specific differentially expressed genes of fungal-inoculated kernels between AF99 and AF32. Color depth represents the degree of significance, as shown in the scale at the right (corrected p-value). Figure S9. Determination of soft-thresholding power (β). a: scale-free topology fit index as a function of the soft-thresholding power, the red line indicates that R2 is equal to 0.85. b: mean connectivity as a function of the soft-thresholding power. Figure S10. The sample dendrogram and trait heatmap. Lines: “0” for AF32 and “1” for AF99; Ino: “0” for mock and “1” for A. flavus inoculation; Time: “0, 0.5, 1.5, 3, 6” indicate the time after inoculation; RC0: “1” for the three biological repetitions of AF99 (mock-treated) at the T0 stage and “0” for other samples; R0: “1” for the three biological repetitions of AF99 (A. flavus inoculation)) at the T0 stage and “1” for other samples; SC0:“1” for the three biological repetitions of AF32 (mock-treated) at the T0 stage and “0” for all the other samples; S0:“1” for the three biological repetitions of AF32 (A. flavus inoculation) at the T0 stage and “0” for all the other samples; the rest are similar as “RC0”, “R0”, “SC0” and “S0”. Figure S11. Scatterplots of gene significance versus module membership for the brown/R0(a), magenta/SC1(b), pink/S1(c), black/S2(d) and brown/S3(e), with correlations and p-values indicated. Figure S12. GO and KEGG pathway analysis of 110 hub genes identified in this study. a. GO analysis of 110 hub genes. Each circle represents an enrichment category, and the size of the circle indicates the number of genes. Detailed enrichment results are shown in Table S9 b. The top 20 associated KEGG pathways for the 110 hub genes. Figure S13. Heat map of 35 genes associated with the hypersensitive response and programmed cell death. Red indicates high expression, blue indicates low expression. Remove very low expression genes (average FPKM< 0.5). [file 12870_2021_2983_MOESM2_ESM.pdf]

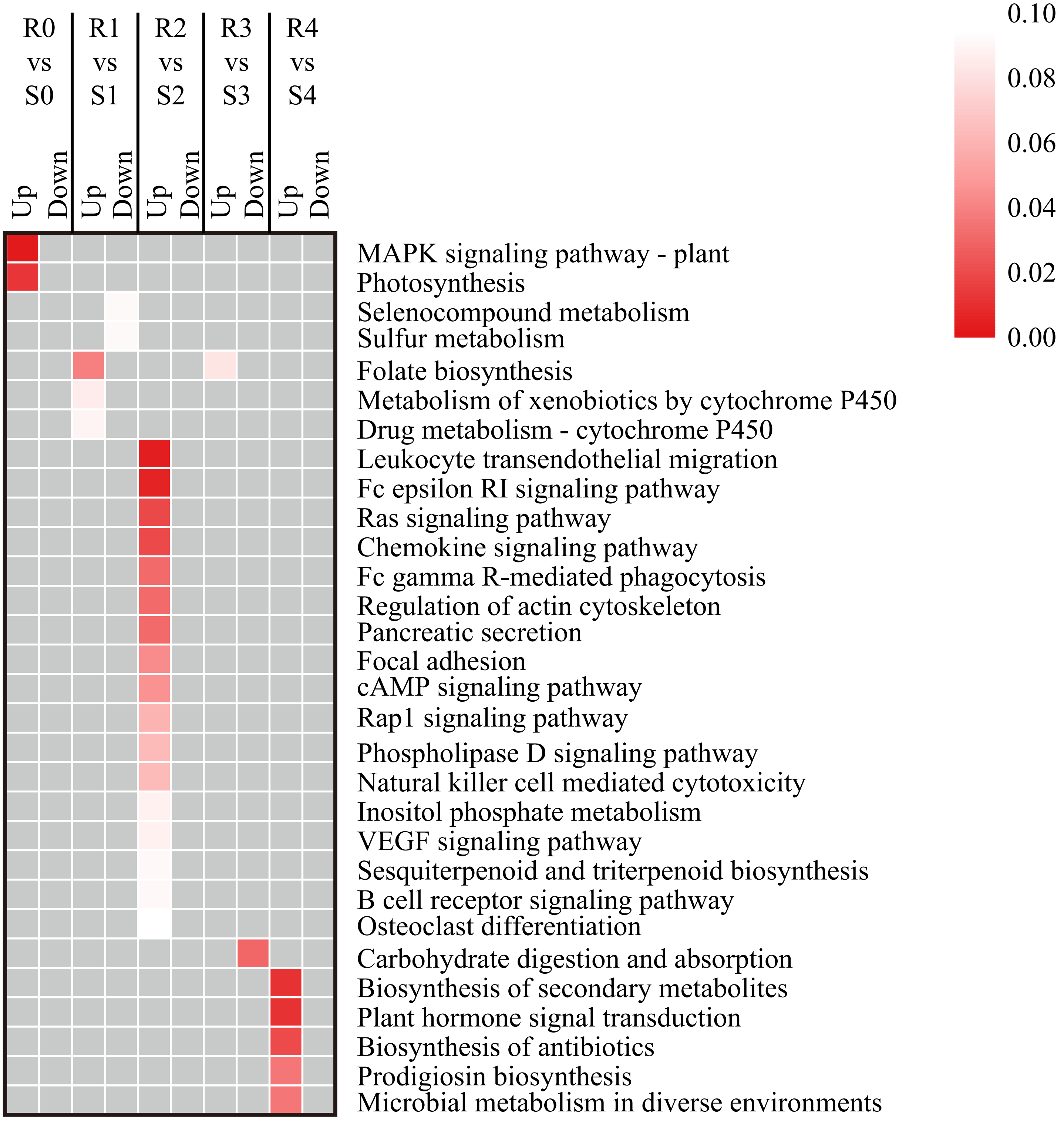

Figure S8. KEGG analysis of the specific differentially expressed genes of fungal-inoculated kernels between AF99 and AF32.
Color depth represents the degree of significance, as shown in the scale at the right (corrected p-value).

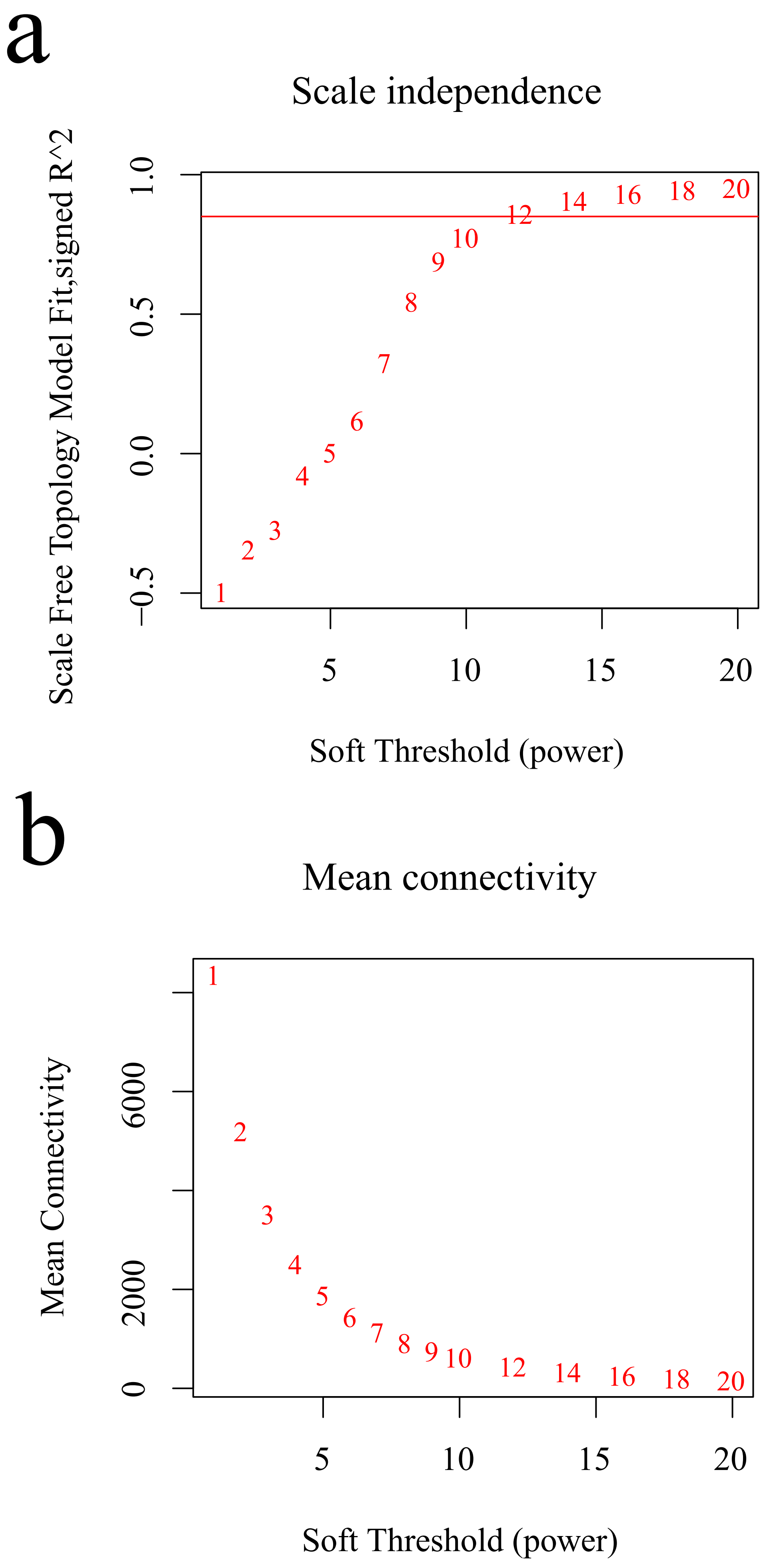

Figure S9. Determination of soft-thresholding power (β).
a: scale-free topology fit index as a function of the soft-thresholding power, the red line indicates that R2 is equal to 0.85. b: mean connectivity as a function of the soft-thresholding power.

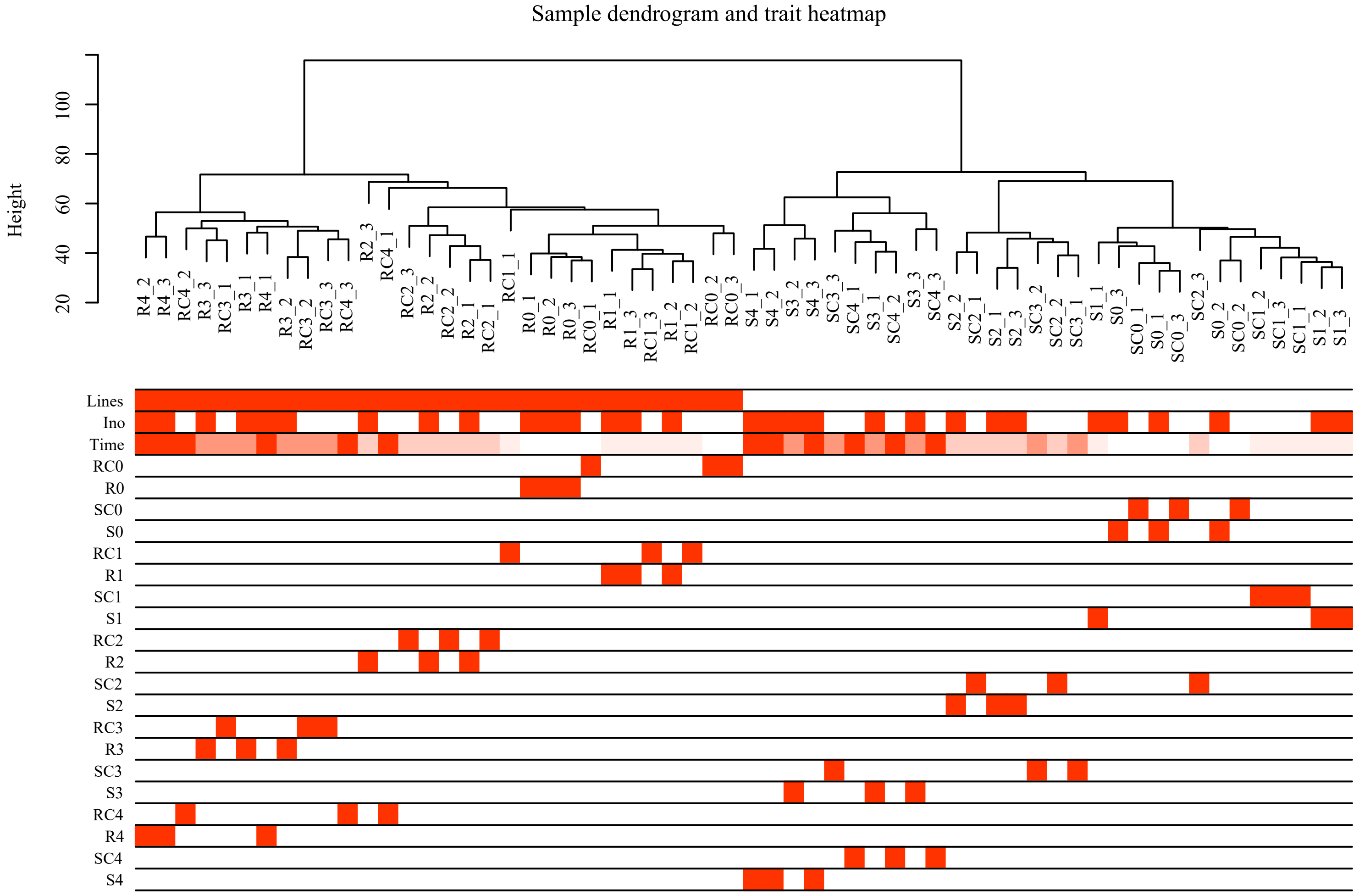

Figure S10. The sample dendrogram and trait heatmap.
Lines: "0" for AF32 and "1" for AF99; Ino: "0" for mock and "1" for *A. flavus* inoculation; Time: "0, 0.5, 1.5, 3, 6" indicate the time after inoculation; RC0: "1" for the three biological repetitions of AF99 (mock-treated) at the T0 stage and "0" for other samples; R0: "1" for the three biological repetitions of AF99 (*A. flavus* inoculation)) at the T0 stage and "1" for other samples; SC0:"1" for the three biological repetitions of AF32 (mock-treated) at the T0 stage and "0" for all the other samples; S0:"1" for the three biological repetitions of AF32 (*A. flavus* inoculation) at the T0 stage and "0" for all the other samples; the rest are similar as "RC0", "R0", "SC0" and "S0".

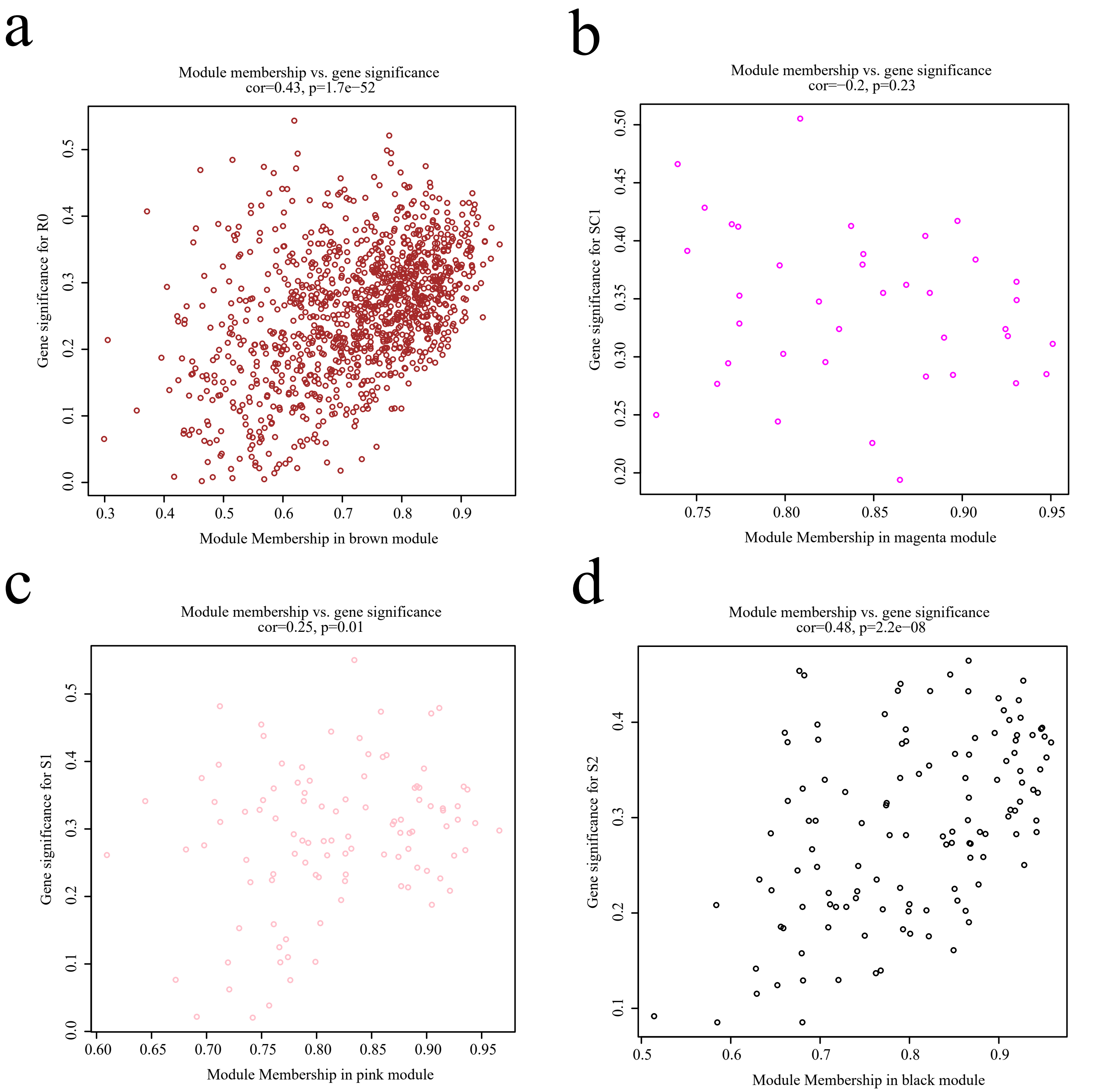

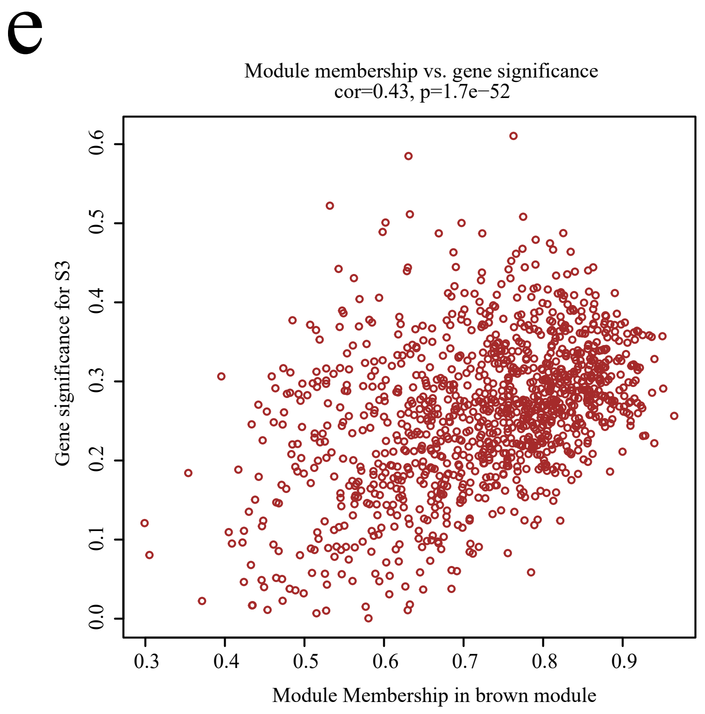

Figure S11. Scatterplots of gene significance versus module membership for the brown/R0(a), magenta/SC1(b), pink/S1(c), black/S2(d) and brown/S3(e), with correlations and p-values indicated.

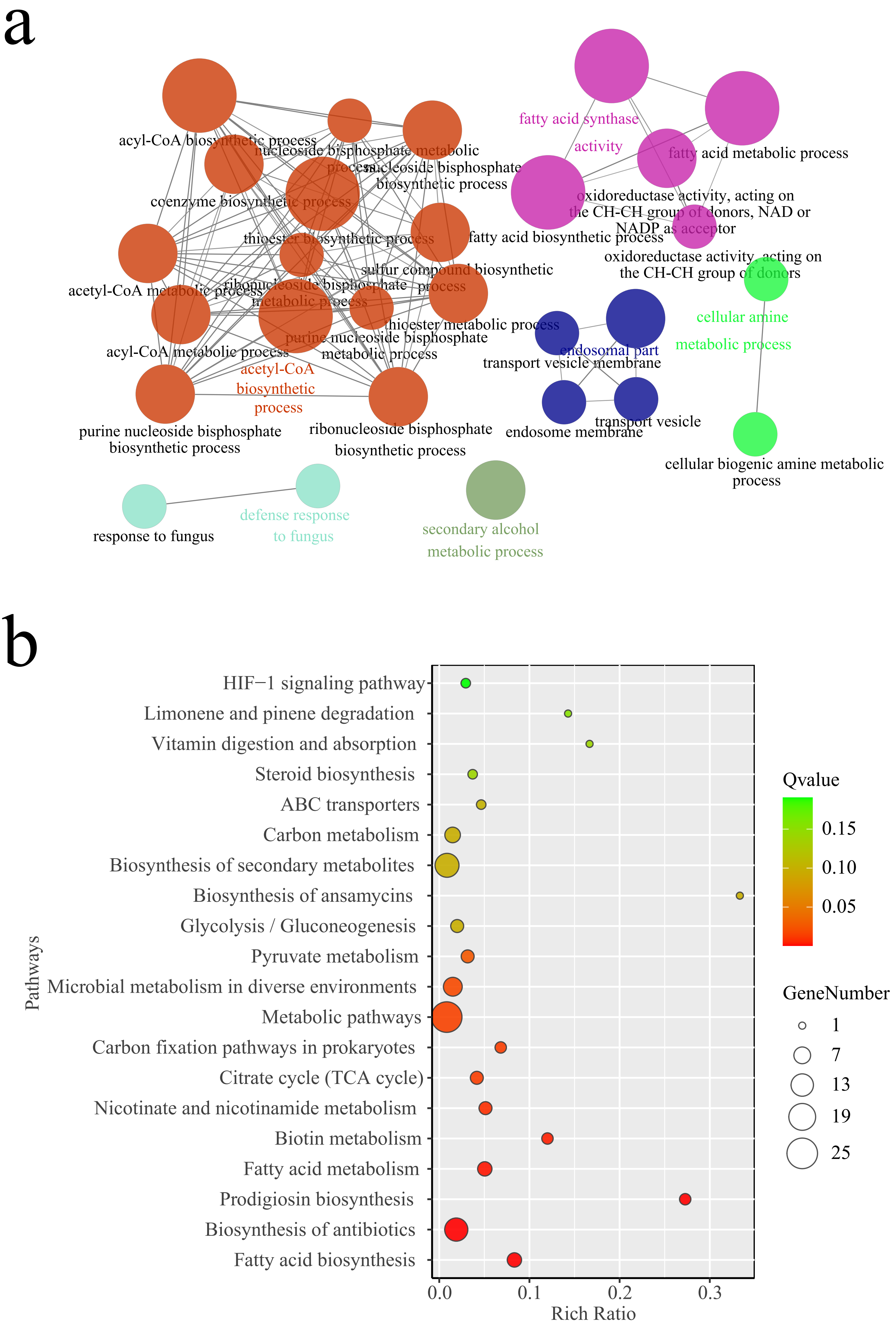

Figure S12. GO and KEGG pathway analysis of 110 hub genes identified in this study.
a. GO analysis of 110 hub genes. Each circle represents an enrichment category, and the size of the circle indicates the number of genes. Detailed enrichment results are shown in Table S9.
b. The top 20 associated KEGG pathways for the 110 hub genes.

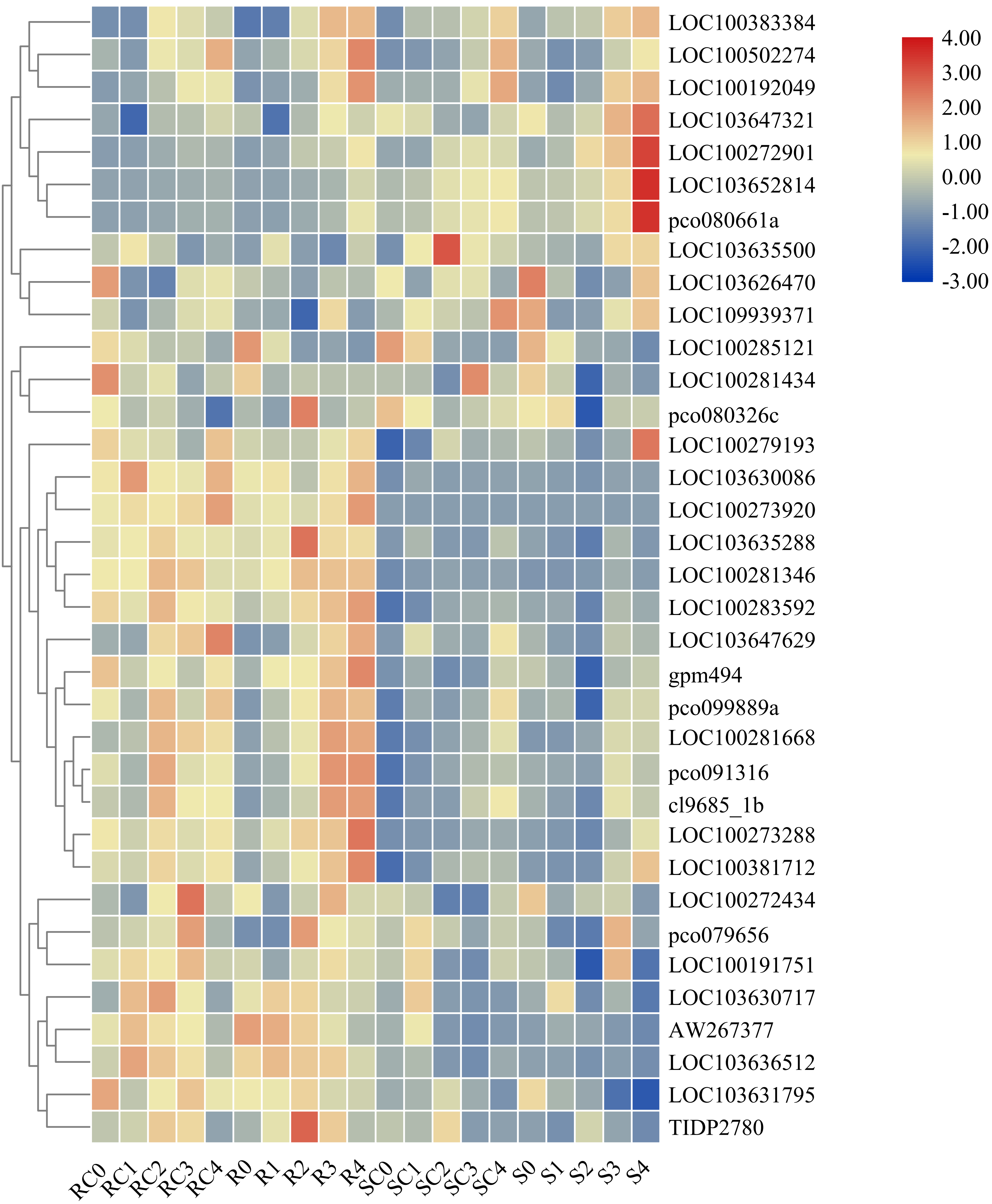

Figure S13. Heat map of 35 genes associated with the hypersensitive response and programmed cell death. Red indicates high expression, blue indicates low expression. Remove very low expression genes (average FPKM<0.5).
